# L_RNA_scaffolder: scaffolding genomes with transcripts

**DOI:** 10.1186/1471-2164-14-604

**Published:** 2013-09-08

**Authors:** Wei Xue, Jiong-Tang Li, Ya-Ping Zhu, Guang-Yuan Hou, Xiang-Fei Kong, You-Yi Kuang, Xiao-Wen Sun

**Affiliations:** 1 The Centre for Applied Aquatic Genomics, Chinese Academy of Fishery Sciences, Beijing 100141, China; 2 College of Fisheries and Life Science, Shanghai Ocean University, Shanghai 201306, China; 3 Key Laboratory of Computational Biology, CAS-MPG Partner Institute for Computational Biology, Shanghai Institutes for Biological Sciences, Chinese Academy of Sciences, Shanghai 200031, China; 4 Heilongjiang River Fisheries Research Institute, Chinese Academy of Fishery Sciences, Harbin 150001, China

**Keywords:** L_RNA_scaffolder, Scaffolding, Transcriptome, Genome

## Abstract

**Background:**

Generation of large mate-pair libraries is necessary for *de novo* genome assembly but the procedure is complex and time-consuming. Furthermore, in some complex genomes, it is hard to increase the N50 length even with large mate-pair libraries, which leads to low transcript coverage. Thus, it is necessary to develop other simple scaffolding approaches, to at least solve the elongation of transcribed fragments.

**Results:**

We describe L_RNA_scaffolder, a novel genome scaffolding method that uses long transcriptome reads to order, orient and combine genomic fragments into larger sequences. To demonstrate the accuracy of the method, the zebrafish genome was scaffolded. With expanded human transcriptome data, the N50 of human genome was doubled and L_RNA_scaffolder out-performed most scaffolding results by existing scaffolders which employ mate-pair libraries. In these two examples, the transcript coverage was almost complete, especially for long transcripts. We applied L_RNA_scaffolder to the highly polymorphic pearl oyster draft genome and the gene model length significantly increased.

**Conclusions:**

The simplicity and high-throughput of RNA-seq data makes this approach suitable for genome scaffolding. L_RNA_scaffolder is available at http://www.fishbrowser.org/software/L_RNA_scaffolder.

## Background

One essential purpose of sequencing a genome is to identify genes for functional study and evolutionary comparison with other species. It requires long genome sequences to predict complete gene structures. The completeness of a genome is usually measured by the N50 statistic, the length such that 50% of the assembled genome lies in blocks of the N50 size or longer. To increase the N50 length, genomic libraries with different inserts are used to span repeat regions and to place contigs in their likely order and orientation in the sequence. This step is repeated from small- to large-insert libraries to generate longer scaffolds. Large-insert libraries are necessary to improve the N50 length [1]. Recently modified clone-based or ligation-based approaches have been developed to generate large mate-pair libraries for Illumina platforms [1-3]. The insert size can be over 10 kb. However, these procedures are complex and time-consuming. Furthermore, in some complex genomes, it is hard to increase the N50 length even with large mate-pair libraries, which leads to low transcript coverage. Thus, it is necessary to develop other simple scaffolding approaches, to at least solve the elongation of transcribed fragments.

When one transcript is not fully covered by a genomic fragment, adjacent exons located in two genomic fragments are used as evidence of linkage between the fragments. This process is similar to how pair-end/mate-pair reads are used in genome scaffolding. Indeed, in the draft human genome, the overlapping fingerprint contigs were first merged with mRNAs and expressed sequence tags (ESTs) [4]. The whole genome shotgun (WGS) strategy is now widely applied into *de novo* genome assembly. It is possible to use transcriptome data alone to scaffold WGS sequences. From the Ensembl annotations of five well-assembled genomes, 4.3%–26.4% of the introns are estimated to be longer than 5 kb (Additional file 1: Figure S1). Although this proportion is low, these large introns cover 42.5%–83.9% of all gene loci (Additional file 1: Table S1). Moreover, mounting evidence suggests that the vast majority of the genome is transcribed [5]. Therefore, transcripts from the pervasively transcribed genomic regions might function as long-insert libraries to scaffold most of the transcribed regions in a genome.

In this study, we aim to employ long transcripts to scaffold genomes. Briefly, our method, L_RNA_scaffolder, seeks to find guide transcript exons, which are anchored to different genomic fragments. An optimal connected fragment is found for every anchored fragment based on the number of transcripts aligned to them. Finally, scaffolding paths are built by walking the optimal connections. We demonstrate that L_RNA_scaffolder provides a high accuracy of genome scaffolding and its performance is better than most scaffolding results by existing leading methods with mate-pair libraries of different inserts. We also show that the improved transcript coverage after scaffolding is close to the complete genome. Finally, we apply our method to the highly polymorphic pearl oyster genome, and demonstrate a significant increase in gene model length. The software is designed to be accessible to a broad audience interested in genome assembly.

## Results

### L_RNA_scaffolder achieves similar transcript coverage to that in the complete genome

**Figure 1:**
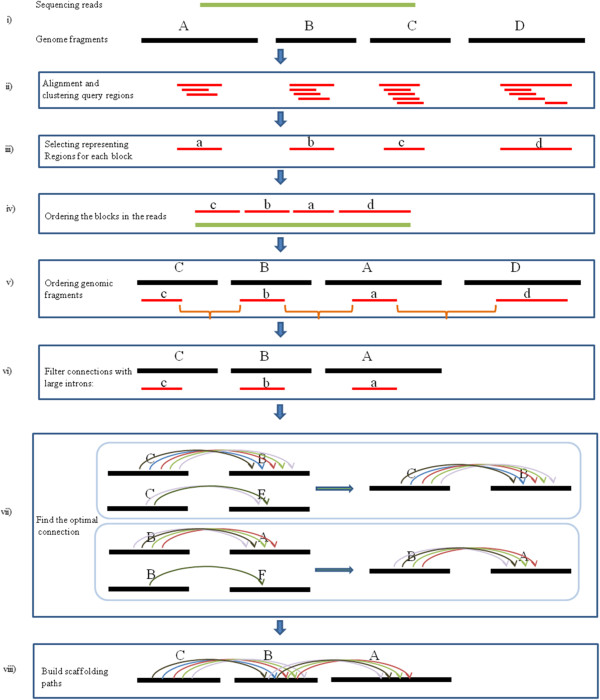
**Schematic diagram of L_RNA_scaffolder steps.** A schematic diagram of L_RNA_scaffolder steps illustrating the selection of ‘guide’ transcripts, ordering the fragments and building paths. **i)** Transcripts (green) are aligned to genomic fragments (black). The genome incompleteness results in that the transcripts are not fully covered and separated into different fragments. **ii)** The transcripts not fully covered are selected as ‘guides’. All alignment query regions are ordered based on their start positions in the read and are clustered into different blocks. **iii)** All query regions are attributed to different blocks. One block is represented by the longest query region in it. **iv)** All blocks are ordered according to their coordinates in the read. **v)** The genome fragments corresponding to the blocks are sorted following block order. **vi)** The DNA sequence between two neighboring blocks is a potential intron (orange). The program filters the fragment connection where the intron is extremely large. **vii)** One fragment might be as the start and/or end in many connections. Every read (arc) stands for supporting evidence connecting the two fragments. The start fragments are assigned to the connection with the most supporting reads. The same is done for the end fragments. One fragment is attributed to at most two connections. **viii)** A scaffolding path consists of at least two fragments, one predecessor and one terminator. Some paths have crossover points. Finally, all fragments are attributed into paths.

To develop L_RNA_scaffolder and to assess its accuracy, we chose zebrafish as the model organism because the genome has been updated recently and well-collected transcriptome data are available. We built scaffolds using L_RNA_scaffolder from 37,298 zebrafish contigs (including clones and WGS contigs) in Zv_9 assembly [6] (1.4 Gb with contig N50 length of 140 kb; hereafter referred to as the initial contigs) with 1.5 million ESTs/mRNAs (940 million bases) (Figure 1).

The pipeline selects ‘guide’ transcripts and their aligned regions using maximal intron length (MIL), minimal length coverage (MLC) and minimal percent identity (MPI) as parameters. We varied the parameters and compared the resulting N50 length. With MLC and MPI set as 0.9, the N50 length increased to the saturation point of 176 kb with MIL over 100 kb (Additional file 1: Figure S2a). To evaluate the influence of MLC, MIL was set as 100 kb and MPI as 0.9. With these parameters, the N50 length was saturated at 177 kb with MLC over 0.95 (Additional file 1: Figure S2b). Finally, for MPI, the N50 length dramatically decreased when the MPI was over 0.9 with the MIL and MLC set as 100 kb and 0.95, respectively (Additional file 1: Figure S2c). This decrease could be the result of genome mutation, RNA editing or sequencing error. Hence, the more stringent the alignment parameters, the fewer usable transcripts that are obtained, leading to a decrease in performance of L_RNA_scaffolder. To obtain the optimal performance of our algorithm for zebrafish genome scaffolding, the MIL, MLC and MPI parameters were set as 100 kb, 0.95 and 0.9, respectively. L_RNA_scaffolder generated 7,366 connections in 3,938 paths where 1,731 of the paths consisted of over two contigs (Additional file 1: Figure S3). These paths consisted of 11,304 contigs, covering a total of 636 Mb (45.4%) of the zebrafish genome. After scaffolding, the N50 size increased from 140 kb to 177.2 kb. Furthermore, the N50 length of the scaffolded fragments increased from 144 kb to 296 kb.

Assuming that the zebrafish reference genome assembly is correct, in order to assess the accuracy of L_RNA_scaffolder, we compared the predicted contig order and orientation of the 7,366 connections to the reference contig connections (Table 1). We found that 5,980 of the 7,366 connections were consistent with the Zv_9 assembly.

**Table 1:** Comparing L_RNA_scaffolder results with Zv_9 assembly

**Type**			**Number**
(i) Consistent	Both order and orientation were consistent		5,980
(ii) Inversions	More evidence for L_RNA_scaffolder^a^		20
	More evidence for Zv_9^b^		0
			20
(iii) Relocations	Correctable relocations		885
	Errant relocations	More evidence for L_RNA_scaffolder	98
		More evidence for Zv_9	21
		Uncertain^c^	102
			1,106
(iv) Translocations	More evidence for L_RNA_scaffolder		43
	More evidence for Zv_9		16
	Uncertain		33
	Contigs from two reference scaffolds		31
	Contigs from one scaffold and one chromosome, respectively		137
			260
Total			7,366

^a^ Three approaches including syntenic block orders, human homolog coverage and zebrafish ‘guide’ transcript completeness, are used to determine the correct connection. The connection with the most evidence is regarded to be correct. For these cases, L_RNA_scaffolder results have more supporting evidence than Zv_9.

^b^ For these cases, Zv_9 connections have more supporting evidence than L_RNA_scaffolder results.

^c^ There is the same amount of evidence or no supporting evidence for both conflicting connections. It is hard to determine which connection is correct.

Following the Genome Assembly Gold Standard Evaluations (GAGE) pipeline [7], the inconsistent connections between L_RNA_scaffolder result and the reference genome were tallied into three types of misjoins, including inversions, relocations and translocations. Twenty predicted connections belong to the inversions, where one contig in the predicted connection is reversed with respect to the reference genome. However, the orientation of the zebrafish transcripts and/or their human homologs supported our predicted orientation of contigs (Additional file 1: Table S2), indicating that these predicted connections were possibly correct.

A total of 1,106 predicted connections were attributed to relocations, where two distant contigs in a reference chromosome were joined together in the predicted assembly. Transcripts span only exonic contigs, leading to that the intronic contigs between them cannot be reconstructed using our method. Indeed, in 885 relocations, the intervals between two contigs in the reference genome were smaller than MIL. Over half of these distances were smaller than 8 kb (Additional file 1: Figure S4). Although the intronic contigs were not scaffolded in these relocations, the predicted connections could recover the complete transcripts and thus were considered as ‘correct’ relocations (see the transcript coverage evaluation below). The other 221 relocations, where two contigs were over MIL apart in the reference genome, were considered as ‘errant’ relocations. Three indicators, including syntenic block order, human homolog completeness, and zebrafish transcript coverage, were used to deduce the correct order for ‘errant’ relocations. One hundred and two relocations did not have any unambiguous supporting indicators (Additional file 2: Table S3); however, the contig orders in 119 relocations could be deduced with clear evidence. L_RNA_scaffolder connections in 98 of these relocations were supported with more evidence compared with that for the reference genome (Additional file 3: Table S4 and Additional file 4: Table S5). This result indicated that two contigs with these connections should be scaffolded together.

A total of 260 connections were attributed to translocations, where two contigs in the predicted assembly were located in two reference chromosomes. For 31 of these translocations, two contigs were from distinct scaffolds in the reference genome, suggesting that these two scaffolds should be joined together. In another 137 translocations, two contigs in the predicted assembly were located in one scaffold and one chromosome, respectively, indicating that this scaffold should be integrated into the reference chromosome. In the remaining 92 translocations, two contigs from different reference chromosomes were scaffolded together in our prediction. We deduced the possibly correct connections for these 92 translocations using the same strategy as for relocations. In 33 translocations, both our predicted assembly and the reference genome had either the same amount of evidence or no supporting evidence and thus the correct connections were hard to determine (Additional file 5: Table S6). In 43 translocations, more evidence supported L_RNA_scaffolder than Zv_9 (Additional file 6: Table S7 and Additional file 7: Table S8).

Overall, assuming that the reference genome is correctly assembled, the corrected accuracy rate of our method is 93.6% (including consistent connections and correctable relocations). If the L_RNA_scaffolder connections in inversions, relocations and translocations with more supporting evidence are also considered as correct, then the corrected accuracy rate of our method reaches 97.6%.

**Figure 2:**
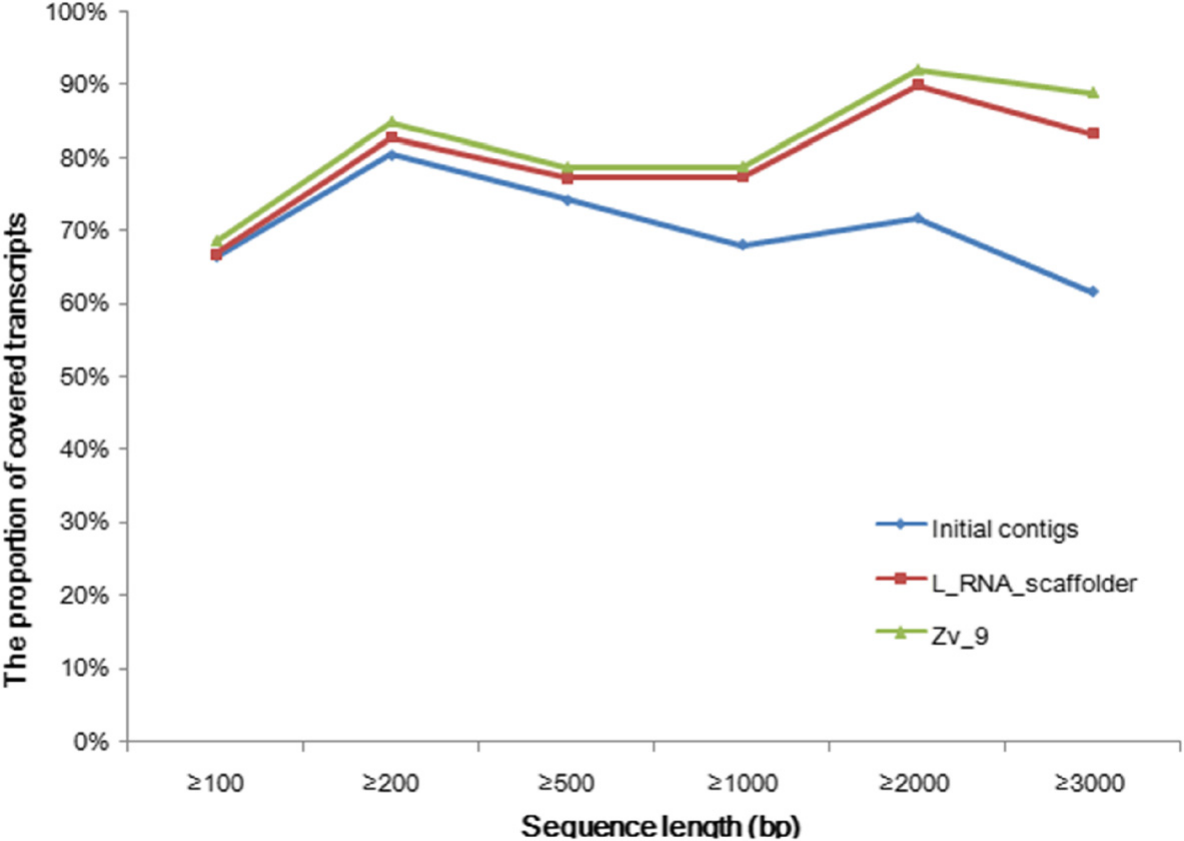
**Comparison of transcript coverage in three zebrafish assemblies.** Initial contigs, the zebrafish clones and WGS sequences; L_RNA_scaffolder, the L_RNA_scaffolder results; Zv_9, the Zv_9 assembly. Cleaned transcripts are aligned to the three zebrafish genomes using BLAT. Identity cutoff is set as 90% and sequence coverage threshold as 90%.

To assess the improvement of transcript coverage by L_RNA_scaffolder, we mapped cleaned zebrafish transcripts to the initial contigs, L_RNA_scaffolder results and Zv_9. We found that the transcript coverage in the L_RNA_scaffolder results was higher than in the contigs and nearly equal to the coverage in Zv_9 (Figure 2). In particular, the coverage for longer transcripts obtained with L_RNA_scaffolder showed better improvement than for short transcripts. For instance, for transcripts longer than 3 kb, the proportion of sequences covered by one scaffold with a length cutoff of 90% increased from 61.5% in the initial contigs to 83.2% after scaffolding. Comparison of the coverage of 248 core eukaryotic genes (CEGs) [8] in the above three genomes showed the same trend (Additional file 1: Figure S5). In Zv_9, most of the contigs are anchored to the 25 chromosomes and therefore the N50 value (final N50 size: 54 Mb [6]) is much longer than L_RNA_scaffolder N50 size. However, a comparison of transcript coverage and CEG coverage between our scaffolds and the Zv_9 assembly demonstrated that L_RNA_scaffolder produced an assembly that had good enough transcript coverage to enable gene prediction.

### Scaffolding the genome with an enlarged transcriptome improves the entire N50 size

Scaffolding the zebrafish genome demonstrated that L_RNA_scaffolder significantly improved transcript coverage, indicating that completeness of the transcribed genome regions increased. Mounting evidence has suggested that the vast majority of the genome is transcribed [5]. Therefore, to estimate whether an enlarged transcriptome could increase the completeness of the entire genome, we scaffolded 27,416 human contigs in the hg19 genome version with 8.8 million human ESTs/mRNAs (4.7 billion bases), a dataset almost six times as large as that of zebrafish. A total of 5,036 paths were built, consisting of 16,661 contigs and covering a total of 1.8 Gb (66.7%) of the human genome. The N50 length of the scaffolded genome increased from 144.2 kb to 432 kb, tripling the initial N50 size. Because transcribed regions occupy the majority of the human genome, the overall N50 length increased from 142 kb to 283.2 kb; a better performance than with the zebrafish data.

**Figure 3:**
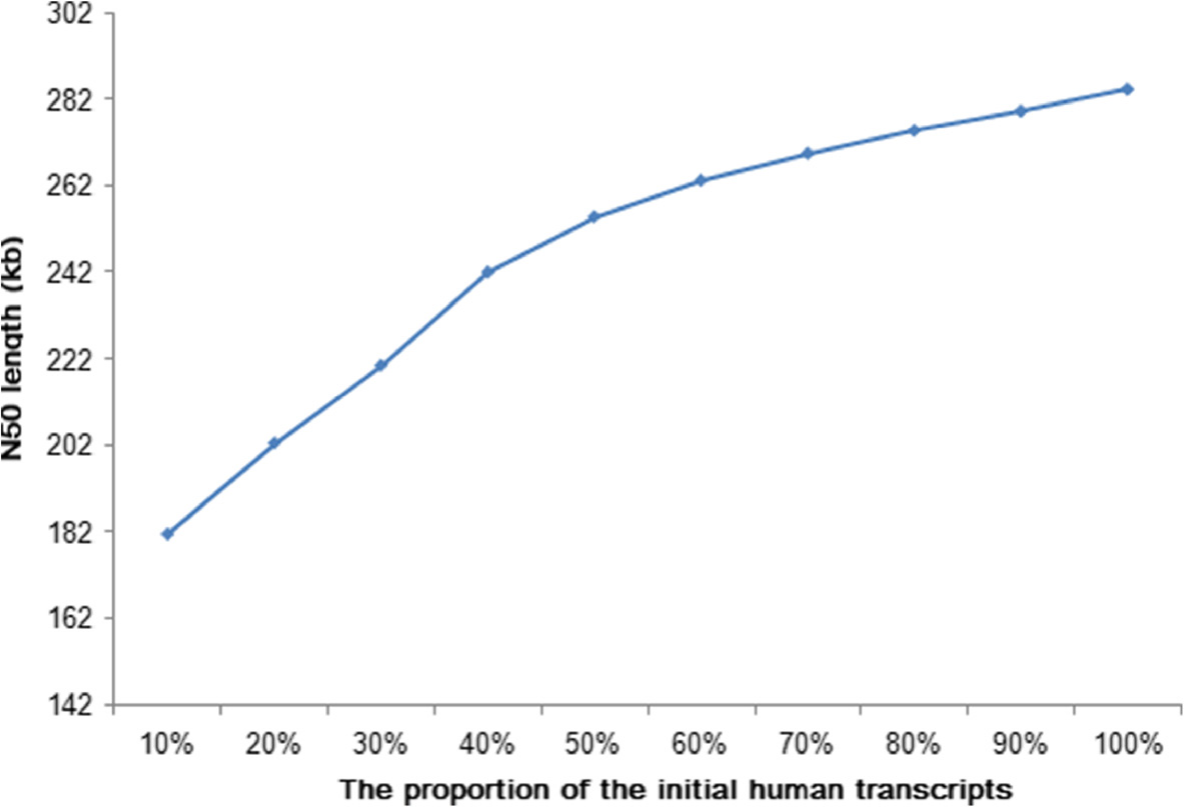
**Effect of human transcript input on N50 length.** Human transcriptome data at defined levels from 10% to 100% are used to produce new assemblies. The N50 length increases but does not reach the saturation point.

We randomly sampled from 10% to 100% of the transcriptome data and used L_RNA_scaffolder to produce new assemblies at each of the defined levels. The N50 increased with more transcriptome data (Figure 3). The N50 was not saturated, indicating that it will improve as more transcriptome data becomes available.

**Figure 4:**
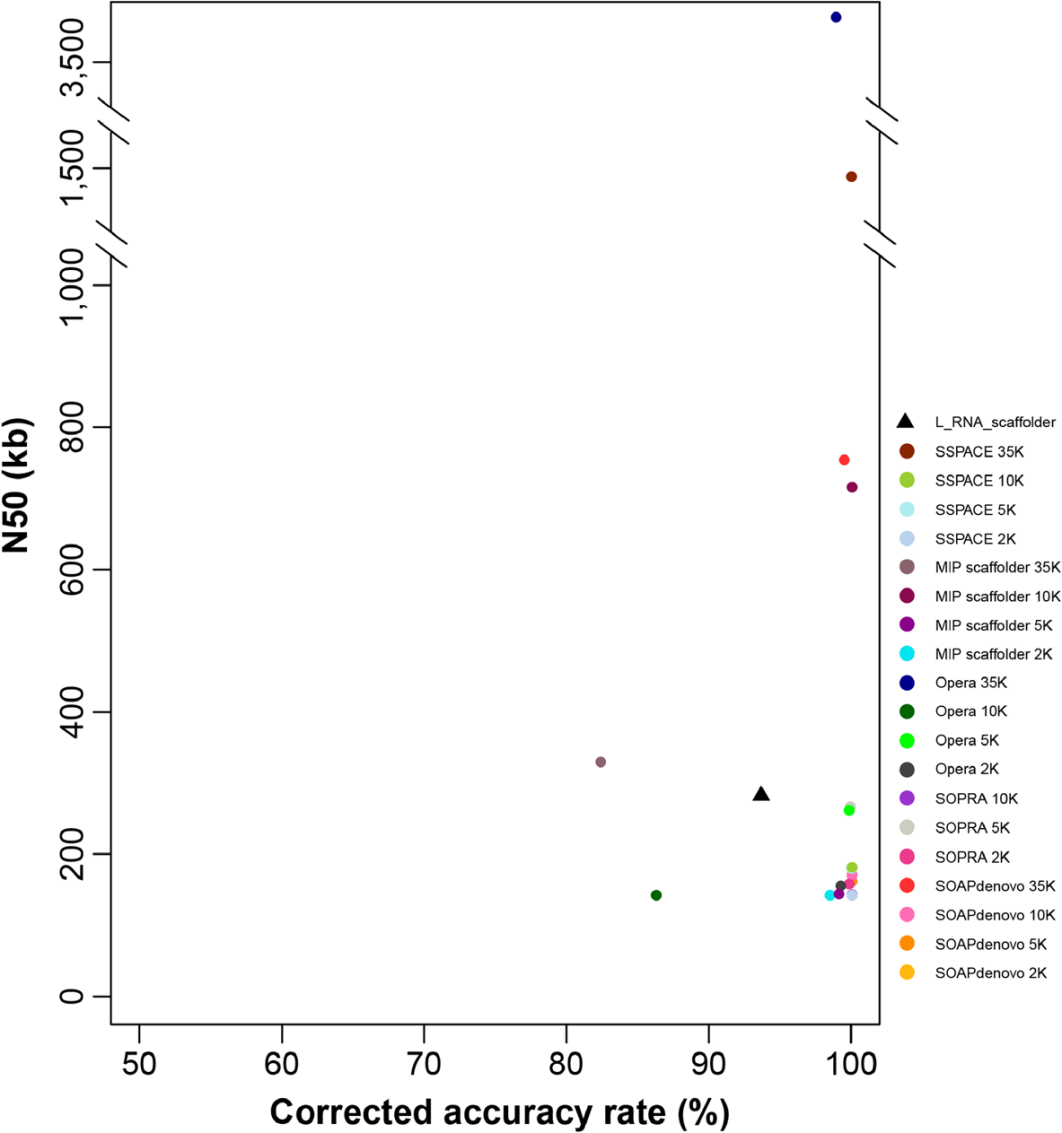
**Corrected accuracy rates versus scaffold N50 values.** The corrected accuracy rate is measured as the proportion of consistent connections and correctable relocations. N50 values represent the size N at which 50% of the genome is contained in scaffolds of length N or larger. The triangle represents the corrected accuracy rate and the N50 value of L_RNA_scaffolder.

Mate-pair libraries are widely used for genome scaffolding. To compare our method’s power with that of existing scaffolding methods, we used five leading scaffolders with four distinct mate-pair libraries (2 kb, 5 kb, 10 kb and 35 kb) to scaffold human genome contigs. The amount of mate-pair reads in all four libraries is equal to the transcriptome data in L_RNA_scaffolder. In Table 2, we present snapshots of the scaffold number and N50 size of 20 scaffoldings. We found that L_RNA_scaffolder produced a larger N50 size than 14 scaffoldings using libraries of 2 kb, 5 kb or 10 kb but the size was smaller than the ones of scaffoldings using the 35 kb library. The N50 size produced by the MIP scaffolder with the 10 kb library was also larger than that of L_RNA_scaffolder (Table 2). Another conclusion was that no scaffolder refrained from misjoin errors, especially relocations and translocations. Following the strategy described above for correcting relocation errors, if the distance between two contigs in the predicted assembly was less than MIL in the reference genome, this relocation was correctable. This produced a revised picture of the scaffolders’ accuracy statistics. As shown in Figure 4 and Table 3, the corrected accuracy of L_RNA_scaffolder was in the range of the five scaffolders.

**Table 2:** Scaffolding the human genome

**Software**	**Library**	**Scaffold number**	**N50 (kb)**
SOAPdenovo	2 kb	27,390	142
	5 kb	22,691	162
	10 kb	21,444	170
	35 kb	10,266	754
SOPRA	2 kb	23,653	158
	5 kb	15,476	266
	10 kb	27,142	143
	35 kb	unfinished, cause unclear	
Opera	2 kb	24,484	155
	5 kb	16,207	261
	10 kb	27,412	142
	35 kb	3,884	3,726
MIP scaffolder	2 kb	27,247	142
	5 kb	26,804	144
	10 kb	8,134	716
	35 kb	13,295	329
SSPACE	2 kb	27,391	142
	5 kb	19,528	180
	10 kb	19,790	181
	35 kb	5,983	1,457
L_RNA_scaffolder		15,792	283

**Table 3:** Statistics on inversions, translocations and relocations in 20 scaffoldings of the human genome

**Software**	**Library**	**Connections**	**Consistent**	**Inversion**	**Translocation**	**Relocation**	
						**Total**	Correctable*
SOAPdenovo	2 kb	6	4	0	0	2	2
	5 kb	4,320	4,196	0	0	124	124
	10 kb	5,456	5,394	0	0	62	62
	35 kb	15,765	14,930	0	82	753	744
SOPRA	2 kb	3,717	3,701	0	0	16	8
	5 kb	11,786	11,665	0	0	121	104
	10 kb	268	236	0	0	32	32
	35 kb	unfinished, cause unclear					
Opera	2 kb	2,917	2,886	0	0	31	7
	5 kb	11,095	10,968	0	0	127	101
	10 kb	29	25	0	0	4	0
	35 kb	23,149	22,058	0	224	872	829
MIP scaffolder	2 kb	129	84	0	1	44	43
	5 kb	537	177	0	3	357	355
	10 kb	12,626	12,452	0	3	171	171
	35 kb	13,297	9,937	1	2,232	1,137	1,019
SSPACE	2 kb	14	11	0	0	3	3
	5 kb	7,871	7,717	0	3	154	154
	10 kb	7,616	7,532	0	0	84	84
	35 kb	21,388	20,473	1	5	912	907
L_RNA_scaffolder		11,579	9,839	3	625	1,123	1,022

Column headers are defined in the main text. *: if the interval between two contigs is smaller than MIL in the reference genome but they are joined together in the predicted assembly, this relocation is considered correct.

**Figure 5:**
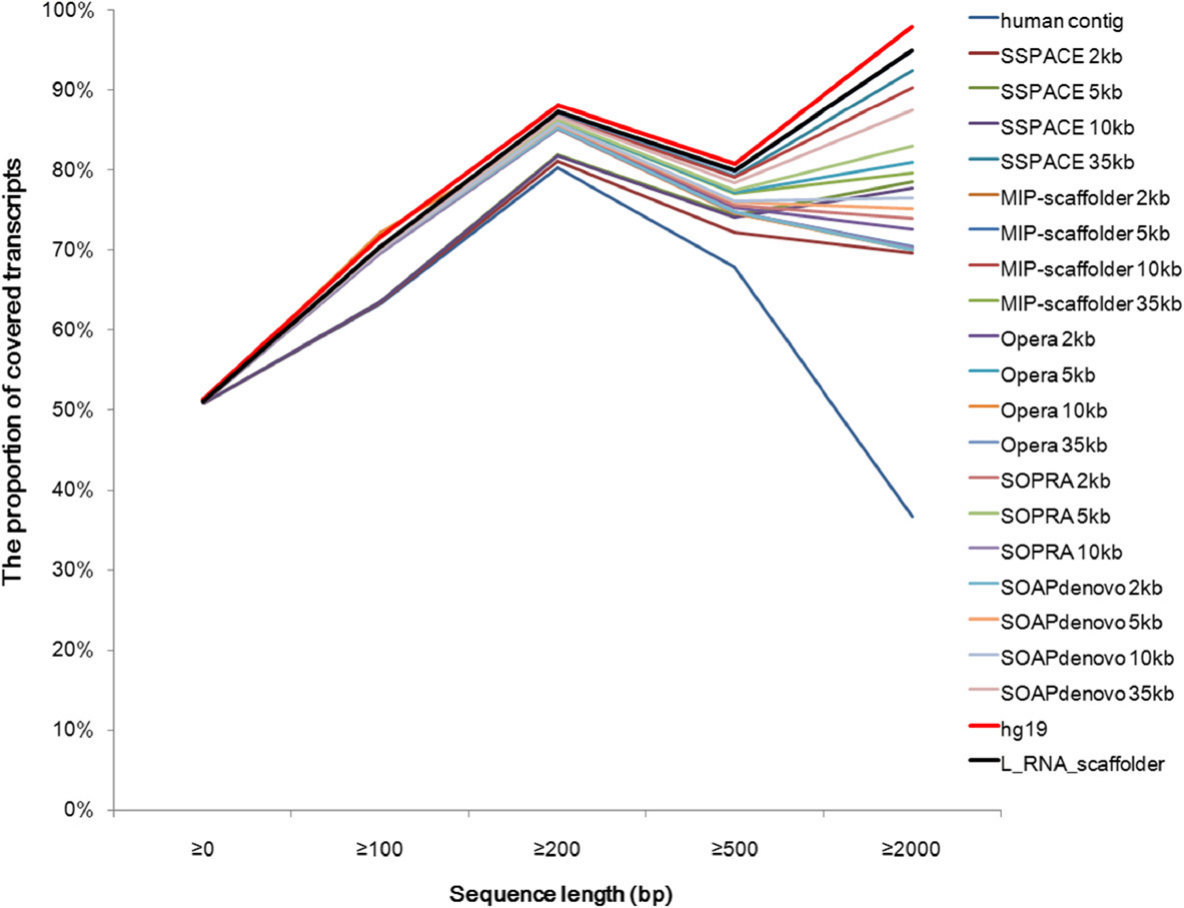
**Comparison of transcript coverage in 20 human genome assemblies by L_RNA_scaffolder and five other scaffolders.** Cleaned transcripts are aligned to 20 scaffolding results of the human genome using BLAT. The parameters are the same as in Figure 2.

Finally, to assess the improvement of transcript coverage with the different methods, we aligned human transcripts against each scaffolding result. As shown in Figure 5, with a length threshold of 90%, the transcript coverage in L_RNA_scaffolder is higher than that in any other scaffolder and is close to the complete human genome. In particular, the coverage of long transcripts (over 2 kb) by L_RNA_scaffolder was dramatically improved. Taken together, these data demonstrate that L_RNA_scaffolder provides not only a practical alternative to the existing scaffolding methods for N50 improvement but also a better solution to improve transcript coverage.

### Scaffolding the draft genome of the highly polymorphic pearl oyster *Pinctada fucata* with the Roche 454 transcriptome

The N50 length of the public genome of the pearl oyster *Pinctada fucata* (1.0 Gb) is only 14.5 kb [9]. Although both the Roche 454 10 kb pair-end library and the Illumina 10 kb library were employed for scaffolding, the N50 size is still small. The major reason for the small N50 was heterozygosity of the genome; nearly two-thirds of the genome is highly polymorphic. For this highly polymorphic genome, we applied L_RNA_scaffolder to assess improvement of both transcript coverage and gene prediction.

**Figure 6:**
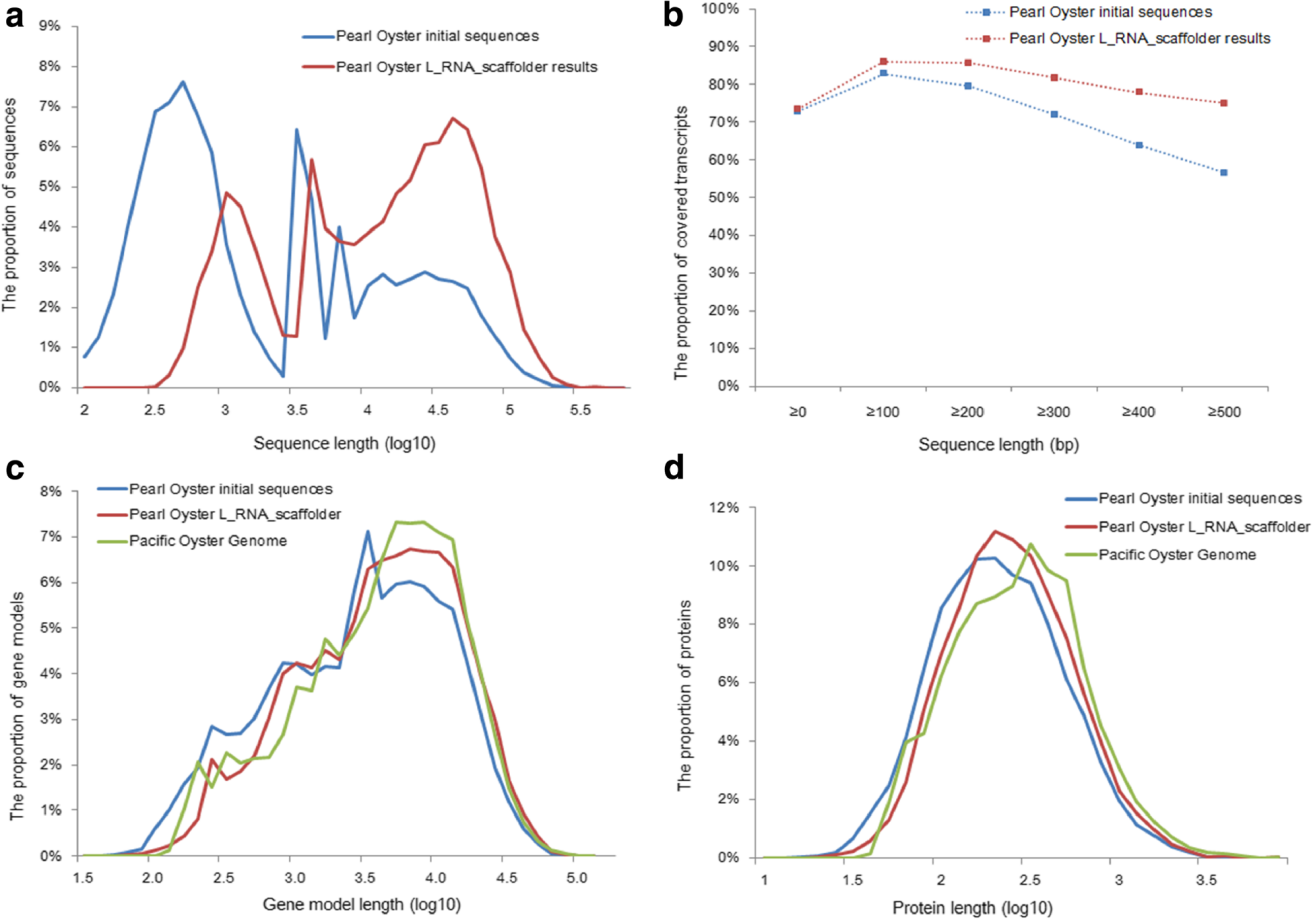
**Scaffolding the pearl oyster genome and gene prediction.** A total of 23,321 initial fragments are joined into 9,206 sequences using L_RNA_scaffolder. **(a)** The length distribution of the initial fragments and scaffolds. **(b)** Transcript coverage in the initial fragments and the 9,206 scaffolds. **(c)** The predicted gene length in the pearl oyster initial fragments, scaffolds and in the Pacific oyster genome. **(d)** The predicted protein length in the pearl oyster initial fragments, scaffolds and in the Pacific oyster genome.

A total of 1.5 million cleaned Roche 454 reads (360 million bases) from polyA(+) transcriptome libraries were used to scaffold the draft genome. L_RNA_scaffolder joined 23,321 initial fragments into 9,206 sequences. These initial sequences accounted for 274 Mb (22.9%) of the pearl oyster genome with an N50 of 48.6 kb. The sequences had biased length distribution; half were shorter than 1 kb. After scaffolding, the N50 length was improved to 62.8 kb. As shown in Figure 6a, this scaffolding mainly merged the fragments shorter than 1 kb into longer sequences. The scaffolding result had a bias towards the long sequences, half of which were longer than 16 kb. The transcript coverage was improved from 76.7% to 82.8%, with the coverage of long reads (over 500 bp) improving considerably from 56.4% to 75% (Figure 6b).

A set of gene models in these sequences was generated using Fgenesh + [10]. The initial sequence contained 17,860 gene models with a median length of 3,467 bp. The products had a median length of 235 amino acids. We predicted 16,605 gene models in the L_RNA_scaffolder results. The median length of the predicted genes was 4,703 bp, much longer than the initial median length (Figure 6c), with a corresponding median protein length of 269 amino acids (Figure 6d). In the genome of the Pacific oyster *Crassostrea gigas*[11], a congener of *P. fucata*, the genes and proteins had median sizes of 5,112 bp and 308 amino acids, respectively. This comparison showed that gene and protein lengths in the L_RNA_scaffolder results were close to those predicted for the Pacific oyster genome (Figure 6c and 6d). Therefore, L_RNA_scaffolder can greatly improve gene completeness for highly polymorphic genomes.

## Discussion

To our knowledge, L_RNA_scaffolder is the first method that uses long-read transcripts for genome scaffolding. Unlike established assembly programs [12,13], L_RNA_scaffolder does not assemble raw reads into contigs and in contrast to existing scaffolding programs that use mate-pair libraries [14], L_RNA_scaffolder uses only transcriptome reads.

A number of transcriptome characteristics provide significant challenges to our scaffolding strategy, making L_RNA_scaffolder more complicated than existing scaffolding programs. First, without anchoring exons, intronic contigs are not scaffolded with exonic contigs, generating correctable relocations. As shown in Table 1, the intervals of two contigs in 80% of relocations (885 out of 1,106) in the Zv_9 assembly are less than MIL in our method. The lost contigs in over half of these relocations are small (Additional file 1: Figure S4). Second, alternative splicing may result in correctable relocations. We carefully examined possible cases where alternative splicing exists in the scaffolds. (1) Both constitutive exons and alternative exons are located in the same contigs and support the same connections. The reconstructed scaffolds could completely cover all alternative splicing variants (Additional file 1: Figure S6a). (2) If one alternative transcript has dominant expression among all transcripts, L_RNA_scaffolder selects this transcript as the guide and builds the connecting paths. If this guide transcript contains all exons of the gene, as shown in Additional file 1: Figure S6b, then L_RNA_scaffolder correctly recovers the connections. Otherwise, the intermediate exonic contigs are not joined, leading to a relocation event (Additional file 1: Figure S6c). To evaluate the coverage completeness of alternatively spliced transcripts in L_RNA_scaffolder result, we used zebrafish spliced variants from the Ensembl database [15] as a test dataset. In the Ensembl database, 12,208 zebrafish genes have 33,924 splicing variants. We aligned these variants to the L_RNA_scaffolder result using BLAT and found that 32,347 variants (95.5%) were completely aligned, indicating that alternative splicing had little influence on our scaffolding. This result is consistent with the transcript coverage in Figure 2. Third, other transcriptome events, including *trans-*splicing [16] and gene fusion [17], generate chimeric RNAs from two different genomic regions and may lead to improper scaffolding, including errant relocations and translocations. If the host transcripts are more abundant than the fusion transcripts, L_RNA_scaffolder selects these host transcripts as the guides and correctly rebuilds the genome (Additional file 1: Figure S7a). If the expression of the fusion transcripts is higher than that of the host transcripts, then L_RNA_scaffolder rebuilds errant relocations or translocations following the guidance of the fusion transcripts (Additional file 1: Figure S7b). In spite of the latter phenomenon, in zebrafish and human genome scaffolds, the errant relocations or translocations (without considering translocations where contigs are located in reference genome scaffolds) have low frequency, accounting for only 4.2% and 6.3%, respectively. Fourth, in our method the gap between two scaffolded contigs is mainly originated from the intron and the intron sizes show the skewed distribution, different from the normal distribution of insert size in mate-pair libraries. Here, to describe the central tendency of intron size distribution, we calculate the median intron size rather than the mean size, which is adopted to measure the insert size distribution in existing scaffolders. Then we estimate the gap size by comparing the median intron value and the distance between two neighboring exons (*D* (n,m), see ‘Filter connections with large introns’ below). Although there are more difficulties in our method than in existing scaffolding programs that obstruct the improvement of assembly coverage and genome continuity, the assessment reveals our method is equivalent in accuracy to these methods. It is noted that assessing our method is on the assumption that the reference genome is correct. For those inconsistent connections, we used three approaches to deduce the correct orders and most of our results were supported.

We have shown that L_RNA_scaffolder is a powerful and effective scaffolder. Compared with existing scaffolding strategies, our method has at least three advantages. First, the simplicity and high throughput of RNA-seq technology could make transcriptome reads widely applicable to genome scaffolding. Mate-pair libraries are very helpful for assemblies; however, these approaches are limited by cloning or ligation efficiency [1-3] and are much more costly than transcriptome sequencing. Our novel approach may have an important impact in the automated reconstruction of genomes. Second, one notable improvement of L_RNA_scaffolder is to increase transcript coverage, which might facilitate gene identification. L_RNA_scaffolder had similar transcript coverage to the complete genomes, making it a convenient tool that can be used to annotate genomes. Third, L_RNA_scaffolder can be applied to highly polymorphic genomes. Increasing numbers of genomes are being published in short fragment form, limiting gene identification. One main reason is high polymorphism, which may split one genomic sequence into separate sets of small scaffolds. Takeuchi *et al.* employed large-insert libraries, including 10 kb mate-pair libraries, to scaffold the pearl oyster genome [9]. However, the N50 size was comparatively small. They estimated that nearly two-thirds of genome sequences were highly polymorphic, which would lead to a small N50 length and incomplete gene structures. With our method, the gene model length in the new genome was close to the length in its congener, demonstrating that L_RNA_scaffolder was suitable for gene prediction in highly polymorphic genomes.

The performance of L_RNA_scaffolder can be improved in several ways with the further development of sequencing technology and alignment tools. First, the transcriptome consists of polyA(−) RNAs and polyA(+) RNAs [18]. Non-ribosomal RNA-seq captures both polyA(−) RNAs and polyA(+) RNAs [19] and will help identify more transcribed genomic regions than polyA(+) RNA-seq. Because genes are spatially and temporally expressed, increased sequencing breadth, including multiple tissues and developmental stages, will also cover more genes. As shown in Figure 3, increasing sequencing depth indeed improved N50 size. The above two approaches would increase sequencing depth and further improve the performance of L_RNA_scaffolder. Second, the remarkable progress in sequencing technologies, especially increasing read length, might improve the performance of our method. L_RNA_scaffolder can directly employ long reads generated from 454 and Ion Torrent sequencers. Read length on the Illumina platform has increased from an initial 30 bp to 250 bp [20]. These reads could be directly aligned to the initial contigs and then employed by our method. Besides, *de novo* assembly of illumina RNA-seq reads has been widely adopted in transcriptome reconstruction [21] and the assembly results are able to function as ‘guides’ to build genome scaffolds (see Additional file 1). The third-generation single-molecule sequencing technologies, for instance, PacBio, significantly improve the read length [22] and have been applied to transcriptome sequencing [23]. In the Additional file 1, we describe the application of a small dataset of PacBio long RNA-seq reads from human brain cerebellum [23] to scaffolding the genome, indicating that our method is also suitable for the third-generation RNA-seq data. Third, L_RNA_scaffolder currently uses the output of BLAT software for scaffolding. The development of a more effective and accurate alignment approach will reduce the error rate and further improve the efficiency of L_RNA_scaffolder.

## Conclusions

L_RNA_scaffolder provides a practical alternative to the existing scaffolding methods. The findings in this paper have been derived using three different practices with different data analysis. The comparison with zebrafish reference genome, combined with order determination, reveals that our algorithm has high accuracy. The promising outcomes with the human genome strongly indicate that long transcript reads can scaffold the genome as effectively as large-insert libraries. Also, we have put emphasis on improving the transcript coverage and gene completeness so that it can be of wide use for gene prediction, even in highly polymorphic genomes. L_RNA_scaffolder can make a significant contribution to the reliable scaffolding of genome assemblies.

## Methods

### Data sources

The zebrafish contigs (including clone sequences and WGS contigs) and ESTs/mRNAs were downloaded from the Ensembl database [15] and UCSC Genome Browser [24], respectively. The human contigs (hg19 version) and ESTs/mRNAs were downloaded from the National Center for Biotechnology Information and UCSC Genome Browser, respectively. The pearl oyster *P. fucata* draft genome was obtained via http://marinegenomics.oist.jp/genomes/downloads?project_id=20. The 454 GS-FLX pearl oyster transcriptome reads were obtained from the NCBI SRA database (Accession: DRX001102, DRX001103, DRX001104 and DRX001105). Here, all the ESTs/mRNAs have been called ‘reads’ , consistent with the concept of next-generation sequencing reads.

### Read processing and alignment

Any vector contamination of reads was removed using SeqClean [25]. For reads produced by 454 or Ion Torrent sequencers, low-quality bases were filtered based on sequencing quality scores. RNA-seq reads from the Illumina platform could be applied by L_RNA_scaffolder in two ways (see Additional file 1). The long transcripts sequenced or assembled were aligned to genomic fragments using BLAT [26].

### Scaffolding overview

The main steps in the L_RNA_scaffolder algorithm can be outlined briefly as:

(a) Screen optimal alignment to identify ‘guide’ reads.

The alignment identity between reads and genomic fragments is calculated using the web-based BLAT percent identity formula in the UCSC Genome Browser [27]. The alignments above a certain MPI are kept for further analysis. The alignment length coverage is calculated as the proportion of aligned length in the whole read. Reads that have alignment lengths above a certain MLC are considered to be fully covered in the genome. All alignments of these reads are then removed. The remaining reads are split into multiple alignment regions and deemed as ‘guides’ that are then used to order and orientate the genomic fragments.

(b) Cluster alignment query regions into ‘blocks’.

All alignment query regions for each ‘guide’ read are ordered based on their start positions in the read. Each query region is compared with all the other regions according to their start positions. The regions that are completely enclosed by other regions are put aside. Next, the distance between any two query regions is measured as the difference between the end positions of the two regions. If the distance is less than a bound constraint (the constraint that represents a bound on the number of nucleotides that separate two regions into different blocks), then these two regions are clustered into one block, otherwise they are classified as two different blocks. Finally, all the query regions are attributed to different blocks. The longest query region in a block is used to represent that block. If one block contains multiple longest query regions, then these regions are likely to originate from repetitive elements or duplicated genes in the genome. This block and the query regions in it are filtered out.

(c) Order and link the ‘blocks’ and their corresponding genomic fragments.

After steps (a) and (b), each remaining block contains only one region aligned to one genomic fragment. One read is then re-built by ordering all the reserved blocks according to their alignment coordinates in it. The corresponding genome fragments are sorted following the block order. If a block is aligned to the minus strand of the genomic fragment, the fragment is reversed. A connection is a directed edge consisting of two genomic fragments. The first fragment is considered as the start and the second fragment is the end. This read is considered as supporting evidence connecting the two fragments.

(d) Filter connections with large introns.

The blocks aligned to genomic fragments are considered as the exons in these fragments and the DNA sequence between two neighboring blocks is a potential intron. For two neighboring blocks (n and m) located in two genomic fragments (*A* and *B*), respectively, *D* (n,m) is defined as the possible intron size between n and m. Then,

$$D\left(n,m\right)\geq \left[\mathrm{Length}\left(A\right)-\mathrm{End}\left(n\right)+\mathrm{Start}\left(m\right)\right].$$

where Length (*A*) is the length of fragment *A*, End (n) is the end position of n in fragment *A* and Start (m) is the start position of m in fragment *B*. Extremely large introns are likely to be a result of misalignment or fusion transcripts [16]. If *D* (n,m) in a read is over a certain MIL, the read is not considered to support the connection of the two fragments.

(e) Find the optimal connection.

If two fragments *A* and *B* are connected through transcripts, we associate the number of the supporting transcripts to the connections, denoted by *{A,B}*. As mentioned in step (c), one fragment might be the start and/or the end in many connections with different evidence numbers. For each fragment *A* as a start, *S* = (*S*_1_,*S*_2_,…,*S*_*N*_), representing the fragments which *A* is connecting. *N* is the number of fragments. We define the optimal connection for *A* as L(*A*).

$$L\left(A\right)=\mathrm{Maximum}\left(\left\{A,S_{1}\right\},\left\{A,S_{2}\right\},\left\{A,S_{3}\right\},\ldots \ldots ,\left\{A,S_{N}\right\}\right).$$

Then, the fragment *A* is designated to L(*A*). For each fragment *B* as an end, *S* = (*S*_1_,*S*_2_,…,*S*_*N*_), representing the fragments which *B* is connected to. We define the optimal connection for *B* as L(*B*).

$$L\left(B\right)=\mathrm{Maximum}\left(\left\{S_{1},B\right\},\left\{S_{2},B\right\},\left\{S_{3},B\right\},\ldots \ldots \left\{S_{N},B\right\}\right).$$

We assign *B* to the optimal connection. If one fragment has two or more connections with the same amount of supporting evidence, this fragment is considered to have no end/start and is discarded. A fusion gene is a chimeric gene generated from two separate genome loci, resulting from relocation or translocation [17]. RNA *trans-*splicing occurs during RNA processing when exons from two different primary RNAs are ligated to a fused RNA [16]. Both gene fusion and *trans-*splicing might lead to improperly merging two fragments into an artificial scaffold. Highly similar homologs might also result in two exons from the homologs being connected together. The process of finding the optimal connection for one fragment decreases the influence of gene fusion, *trans-*splicing or homologs on the scaffolding algorithm. The reserved fragments are then attributed to two connections at most and classified into three types: (i) crossover points where the fragment is the start in one connection and the end in another connection; (ii) predecessors where the fragment only exists in one connection and functions as the start; and (iii) terminators where the fragment only exists in one connection at the end.

(f) Build scaffolding paths by walking the optimal connections.

Select one predecessor and search for a crossover point from the optimal connections. Then search for a new crossover point for the prior crossover point. Repeat these searches to extend the scaffolding path until one terminator is reached. After all the predecessors are walked, all the fragments are attributed into scaffolding paths.

(g) Estimate gap size from intron size distribution.

The gap between two exonic contigs is mainly from the intron. As shown in Additional file 1: Figure S1, the intron sizes show skewed distribution and most are small. We plot the size distribution of introns from the transcripts that are fully covered in the initially contigs and estimate the median intron size. Then, if *D*(n,m) is smaller than the median value, we insert a sequence composed of letter ‘N’ , the number of which is decided by comparing *D*(n,m) and the median intron size. Otherwise, 100 Ns are inserted between two contigs just to indicate a possible gap.

### Assessment of scaffolding accuracy

The accuracy of L_RNA_scaffolder is mainly examined by measuring the number of misjoin errors that are defined in the GAGE pipeline [7]. Assuming that the reference genome is correctly assembled, we compare the contig order and orientation between the predicted assembly and the reference genome. A misjoin error is an event where two sequences are joined together in the assembly in a manner that is inconsistent with the reference. These misjoins are tallied into inversion events, relocations, and translocations. An inversion is a switch between strands (and orientation). Relocations connect distant contigs from the same chromosome. Translocations connect segments from different chromosomes. Details of how we compare the predicted assembly and the reference genome are in the Additional file 1.

Our scaffolding method focuses on scaffolding exonic contigs and therefore intronic contigs between exonic contigs are possibly lost, leading to a relocation event. For example, suppose that contigs {*A, B, C*} are located in the reference genome, where *A* and *C* are exonic contigs and *B* is an intronic contig. L_RNA_scaffolder might reconstruct an {*A, C*} connection while missing out contig *B*. Because this connection also recovers the full transcript, we consider this event as a correctable relocation. As shown in Additional file 1: Figure S1, few introns are larger than 100 kb, the MIL set in our method. Thus, if *A* and *C* are less than MIL apart in the reference genome but joined together in the predicted assembly, this relocation is also considered correct. Otherwise, it is considered as an errant relocation. This provides revised scaffolder contiguity statistics. Finally, the corrected accuracy rate = 1 - (inversions + errant relocations + translocations) / total connections.

The above accuracy assessment is on the basis of the assumption that the reference genome is correctly assembled. For the inversions, errant relocations and translocations, to determine which connection is correct, we use three approaches, syntenic block order, human homolog coverage and zebrafish ‘guide’ transcript completeness. Details of how the correct connection is determined are in the Additional file 1.

### Performance comparison with existing scaffolding methods using mate-pair libraries of different sizes

To evaluate the performance of our method compared with existing scaffolding methods, we scaffold human genome contigs (hg19 version) using our method and five leading genome scaffolders, including SSPACE [14], SOAPdenovo [28], Opera [29], MIP scaffolder [30] and SOPRA [31]. All of these utilize mate-pair Illumina libraries and are open-source assemblers. Short reads from four mate-pair Illumina libraries, including 2 kb, 5 kb, 10 kb and 35 kb, are downloaded from the SRA database (NCBI Accession: SRX176510). The SolexaQA package [32] are used to filter out low-quality bases using the default parameters. We extract the same amount of paired reads as the data size in L_RNA_scaffolder (8.8 million) for further scaffolding. For each method, we run scaffolding using the default parameters. Details of running five scaffolders are given in the Additional file 1.

The performance of these five scaffolding methods is also examined by measuring the N50 value and the number of misjoin errors, defined in the GAGE pipeline [7]. We further compute the transcript coverage of each scaffolding result. ESTs/mRNAs are aligned against the scaffolded genomes. If the sequence coverage of one transcript is over 90%, then we consider it fully covered by the genome.

### Software availability

L_RNA_scaffolder and the comparison with other scaffolders, including the raw reads and 20 scaffolding results, are freely available at http://www.fishbrowser.org/software/L_RNA_scaffolder.

## Abbreviations

ESTs: Expressed sequence tags; WGS: Whole genome shotgun; MIL: Maximal intron length; MLC: Minimal length coverage; MPI: Minimal percent identity.

## Competing interests

The authors declare that they have no competing interests.

## Authors’ contributions

JT Li and W Xue wrote the paper. JT Li and WX Sun directed and coordinated the research. W Xue and JT Li developed and maintained L_RNA_scaffolder program. W Xue scaffolded the zebrafish and human genomes. JT Li ran five leading scaffolders, developed the assessment methods and supervised the assessment. W Xue, YP Zhu, GY Hou and XF Kong performed comparative genome assessment, transcriptome coverage and homolog completeness. YY Kuang predicted the gene models of *P. fucata*. All authors read and approved the final manuscript.

## Supplementary Material

Additional file 1: Figure S1 Intron distribution in human, mouse, cow, chicken and medaka. **Figure S2**: Comparisons of the performance of L_RNA_scaffolder with different parameters. **Figure S3**: The contig number in zebrafish scaffolding paths. **Figure S4**: The length distribution of lost contigs in the correctable relocations. **Figure S5**: The coverage of core eukaryotic genes in three versions of zebrafish genome. **Figure S6**: Alternative splicing may influence L_RNA_scaffolder accuracy. **Figure S7**: *Trans-*splicing and gene fusion may influence L_RNA_scaffolder accuracy. **Table S1**: The proportion of long introns in transcribed genomic regions. **Table S2**: Twenty inversions in zebrafish genome scaffolding. Click here for file

Additional file 2: Table S3 One hundred and two uncertain ‘errant’ relocations. Click here for file

Additional file 3: Table S4 Ninety eight ‘errant’ relocations where L_RNA_scaffolder connections had more supporting evidence. Click here for file

Additional file 4: Table S5 Twenty one ‘errant’ relocations where Zv_9 reference connections had more supporting evidence. Click here for file

Additional file 5: Table S6 Thirty three uncertain translocations. Click here for file

Additional file 6: Table S7 Forty three translocations where L_RNA_scaffolder connections had more supporting evidence. Click here for file

Additional file 7: Table 8 Sixteen translocations where Zv_9 reference connections had more supporting evidence. Click here for file
