# Correction to: L_RNA_scaffolder: scaffolding genomes with transcripts

**DOI:** 10.1186/s12864-019-5856-1

**Published:** 2019-06-07

**Authors:** Wei Xue, Jiong-Tang Li, Ya-Ping Zhu, Guang-Yuan Hou, Xiang-Fei Kong, You-Yi Kuang, Xiao-Wen Sun

**Affiliations:** 1 The Centre for Applied Aquatic Genomics, Chinese Academy of Fishery Sciences, Beijing, 100141 China; 2 College of Fisheries and Life Science, Shanghai Ocean University, Shanghai, 201306 China; 3 Key Laboratory of Computational Biology, CAS-MPG Partner Institute for Computational Biology, Shanghai Institutes for Biological Sciences, Chinese Academy of Sciences, Shanghai, 200031 China; 4 Heilongjiang River Fisheries Research Institute, Chinese Academy of Fishery Sciences, Harbin, 150001 China

**Correction to: BMC Genomics**

**https://doi.org/10.1186/1471-2164-14-604**

Following the publication of this article [1], the authors reported that the link to the software described in the article is no longer valid. They have therefore provided the following alternative address in this Correction article in order to gain access to the software: https://github.com/CAFS-bioinformatics/L_RNA_scaffolder
