# Supplementary material for: L_RNA_scaffolder: scaffolding genomes with transcripts
Source: BMC Genomics. 2013 Sep 8;14:604. doi: 10.1186/1471-2164-14-604 (PMC3846640; doi:10.1186/1471-2164-14-604)
Supplement: Additional file 1: Figure S1 — Intron distribution in human, mouse, cow, chicken and medaka. Figure S2: Comparisons of the performance of L_RNA_scaffolder with different parameters. Figure S3: The contig number in zebrafish scaffolding paths. Figure S4: The length distribution of lost contigs in the correctable relocations. Figure S5: The coverage of core eukaryotic genes in three versions of zebrafish genome. Figure S6: Alternative splicing may influence L_RNA_scaffolder accuracy. Figure S7: Trans-splicing and gene fusion may influence L_RNA_scaffolder accuracy. Table S1: The proportion of long introns in transcribed genomic regions. Table S2: Twenty inversions in zebrafish genome scaffolding. [file 1471-2164-14-604-S1.pdf]

L_RNA_scaffolder: scaffolding genomes with transcripts

Wei Xue, Jiong-Tang Li, Ya-Ping Zhu, Guang-Yuan Hou, Xiang-Fei Kong, You-Yi Kuang and Xiao-Wen Sun

Supplementary Methods

Recipes for human genome scaffolding

Here we describe how we run each scaffolder on human genomes in this study. Note that we run each scaffolder with the default parameters. Although better results may be obtained with different parameters, the default parameters should provide a good starting point. At our website, http://www.fishbrowser.org/software/L_RNA_scaffolder, we have placed the raw mate-pair libraries, the initial contigs and all the assemblies. These recipes allow others to replicate our results.

The file of human contigs is named as 'contigs.fa'. For each scaffolder, we take the 2 kb mate-pair library as a practical instance. The fastq files of 2 kb mate-pair library are 2K_1.fastq and 2K_2.fastq.

(1) To run SOAPdenovo, we make a configure file, named as *soapdenovo.lib*.

```
max_rd_len=100
[LIB]
avg_ins=2000
reverse_seq=1
asm_flags=2
rank=1
pair_num_cutoff=2
q1=2K_1.fastq
q2=2K_2.fastq
```

Then we run the following commands:

```
prepare -c  contigs.fa -K 24 -g EC
SOAPdenovo31mer map -s soapdenovo.lib  -g EC
SOAPdenovo31mer scaff -g EC
```

(2) For **SOPRA**, we format two fastq files into a fasta file (named as srr.fasta) which the program requires. The following commands are used:

perl s_prep_contigAseq_v1.4.6.pl -contig contigs.fa -mate srr.fasta -a outdir

bowtie-build contigs_sopra.fasta SOPRA

bowtie -v 0 -m 1 -f --sam SOPRA srr_sopra.fasta > mysam_mate_illu1

perl s_parse_sam_v1.4.6.pl -sam mysam_mate_illu1 -a outdir

perl s_read_parsed_sam_v1.4.6.pl -parsed mysam_mate_illu1_parsed -d 2000 -a outdir

perl s_scaf_v1.4.6.pl -w 5 -o orientdistinfo_c5 -a outdir

(3) For **SSPACE**, we generate a library file, named as libraries.txt.

Lib1 2K_1.fastq 2K_2.fastq 2000 0.125 RF

We run the command:

perl SSPACE_Basic_v2.0.pl -l libraries.txt -s contigs.fa -k 5 -x 0 -z 0 -a 0.7 -n 15 -T 1 -p 0 -b 2K

(4) To run **Opera**, two fastq files are converted into two fasta files, labeled as lib_1_1.fa and lib_1_2.fa, respectively. Then the following command is run:

perl preprocess_reads.pl contig.fa lib_1_1.fa lib_1_2.fa lib_1.map bowtie

opera contigs.fa lib_1.map results

(5) For **MIP scaffolder**, reads in the Illumina mate pairs are renamed and the appropriate ends are reversed into SOLiD format following the requirement of this package. The new fastq files are R3.fq and F3.fq. We specify the following parameters in the configure file (2K.config).

genome_length=3000000000

minimum_support=2

mappings= filter.merged.sam

orientation=SOLID

insert_length=2000

min_insert_length=1800

max_insert_length=2200

Additionally in the coverage file the coverage of each contig is set as 1. The commands are:

bowtie-build contigs.fa CONTIG

bowtie -v 0 -m 1 --sam CONTIG R3.fq >R3.sam

bowtie -v 0 -m 1 --sam CONTIG F3.fq >F3.sam

perl merge-mapping-lines.pl F3.sam R3.sam merged.sam

env LC_COLLATE=C sort -t$'\t' -k 3,3 merged.sam > merged.sam.sorted1

env LC_COLLATE=C sort -t$'\t' -k 14,14 merged.sam > merged.sam.sorted2

rm merged.sam

filter-mappings.sh merged.sam.sorted1 merged.sam.sorted2 filter.merged.sam

perl mip-scaffolder.pl 2K.config contigs.fa coverage.file ./

Accuracy Assessment following the GAGE pipeline

To compute scaffold correctness in zebrafish and human genome assembly, firstly the contigs in one predicted scaffold are sorted on the reference genome following GAGE pipeline [1]. Briefly, the scaffolds are split into smaller contigs (S_1,S_2,...,S_N) whenever a gap is encountered. These contigs are numbered sequentially in increasing order, generating (N-1) predicted connections (S_i, S_{i+1}) consisting of two sequentially contigs. These contigs are mapped to the reference genome using nucmer [2]. Show-tiling is run to generate the order of contigs on the reference genome, forming multiple reference connections |S_i, S_{i+k}| ($k \neq 0$). Secondly, we compute the correctness by comparing the predicted connections and reference connections. For one contig *i*, if k in the reference connection is equal to 1 and the orientation of S_{i+1} in the two connections are the same, then the predicted connection is consistent with the reference connection. Otherwise, it is defined as a misjoin error, where two contigs are joined together in the assembly in a manner that is inconsistent with the reference

genome. These misjoins are tallied into inversions, relocations, and translocations. An inversion is a switch between strands (and orientation), where k is equal to 1 but the orientations of S_{i+1} in the two connections are opposite. In one relocation, k is not equal to 1 and both S_i and S_{i+1} are from the same chromosome. The predicted assembly connects two contigs S_i and S_{i+1} from different chromosomes, defined as a translocation event. Our scaffolding method focuses on scaffolding exonic contigs and therefore intronic contigs between two exonic contigs is possibly lost, leading to a relocation event. For example, suppose that contigs (S_{i-1}, S_i, S_{i+1}) are located in the reference genome, where S_{i-1} and S_{i+1} are exonic contigs and S_i is intronic contig. L_RNA_scaffolder might reconstruct an (S_{i-1}, S_{i+1}) connection while contig S_i is missing. If S_{i-1} and S_{i+1} are less than MIL apart in the reference genome but joined together in the predicted assembly, this relocation is also considered correct. Otherwise, it is considered as an errant relocation. The strategy is also applied into the accuracy assessment for the five existing scaffolders. Finally, we count the corrected accuracy rate of each scaffolding result. The corrected accuracy rate = 1 - (inversions + errant relocations + translocations) / total connections.

Determining correct connections

The accuracy assessment is on the basis of the reference genome is correct. To determine whether the errant relocations and translocations in zebrafish genome scaffolding are correct, we calculate the supporting evidence numbers for L_RNA_scaffolder connections and Zv_9 connections using three approaches, syntenic block order, human homologs coverage and zebrafish 'guide' transcript completeness. First, zebrafish syntenic blocks in five genomes, Medaka (oryLat2), Stickleback (gasAcu1), Tetraodon (tetNig2), Fugu (fr3) and human (hg19), are converted using the liftOver program [3] and the genome-wide pair-wise alignments between zebrafish and these species is downloaded from the UCSC Genome Browser. If the corresponding syntenic blocks to two

linked contigs in L_RNA_scaffolder are adjacent in one of the above genomes, this is considered to be supporting evidence for this linkage. The same procedure is followed for the connections in Zv_9. Second, homologs should be covered as much as possible by the genome and homolog coverage is a common indicator of the quality of a genome assembly [4]. The alignment of human proteins to zebrafish contigs, mapped by chained tBLASTn, is downloaded from the UCSC Genome Browser. Better homolog coverage is another piece of supporting evidence for a connection. Third, if a connection has better coverage of a 'guide' transcript, then this transcript is the third supporting evidence for this connection. Finally, if L_RNA_scaffolder connections and Zv_9 connections have the same amount of evidence, it is hard to determine which connections are correct; otherwise, the connection with the most evidence is regarded to be the correct one.

Scaffolding human genome with Illumina RNA-seq data

We scaffold human genome contigs with illumina RNA-seq data in two ways. Two datasets of 100 bp RNA-seq data from polyA(+) RNA libraries are downloaded from NCBI SRA database (SRR324684 and SRR324685). In the first way, the fastq sequences are converted to fasta format and aligned to human contigs using BLAT. The alignments are input into L_RNA_scaffolder for scaffolding. The N50 length increases to 163.5 kb. In the second way, RNA-seq data is firstly assembled using Trinity [5]. The *de novo* assembled transcripts are aligned using BLAT and the output guides genome scaffolding. The N50 length reaches 159.5 kb. These scaffoldings demonstrate that L_RNA_scaffolder is suitable for illumina RNA-seq data.

Scaffolding human genome with Pacbio RNA-seq data

PacBio, a third generation sequencing (TGS) technology, generates longer reads than the second-generation sequencing platforms. The PacBio platform can yield reads of average length over 2,500 bp and some reads are over 10 kb [6]. However, these reads have high error rate (up to 15%). To demonstrate that our method is

applicable to PacBio RNA-seq data, 174,246 PacBio long RNA-seq reads from human brain cerebellum [6] are used to scaffold human genome with our method. Considering that currently the reads have high error rate, the alignment identity is set as 80%. After scaffolding, the N50 reaches 146.8 kb. Although the read amount is much small, the N50 value is larger than the ones of the scaffoldings using 2 kb mate-pair library by SOAPdenovo, Opera, MIP scaffolder or SSPACE. This practice indicates that our method has the practical impact on genome scaffolding using the novel single-molecule sequencing technologies.

Supplementary Figures

Supplementary Figure 1. Intron distribution in human, mouse, cow, chicken and medaka.

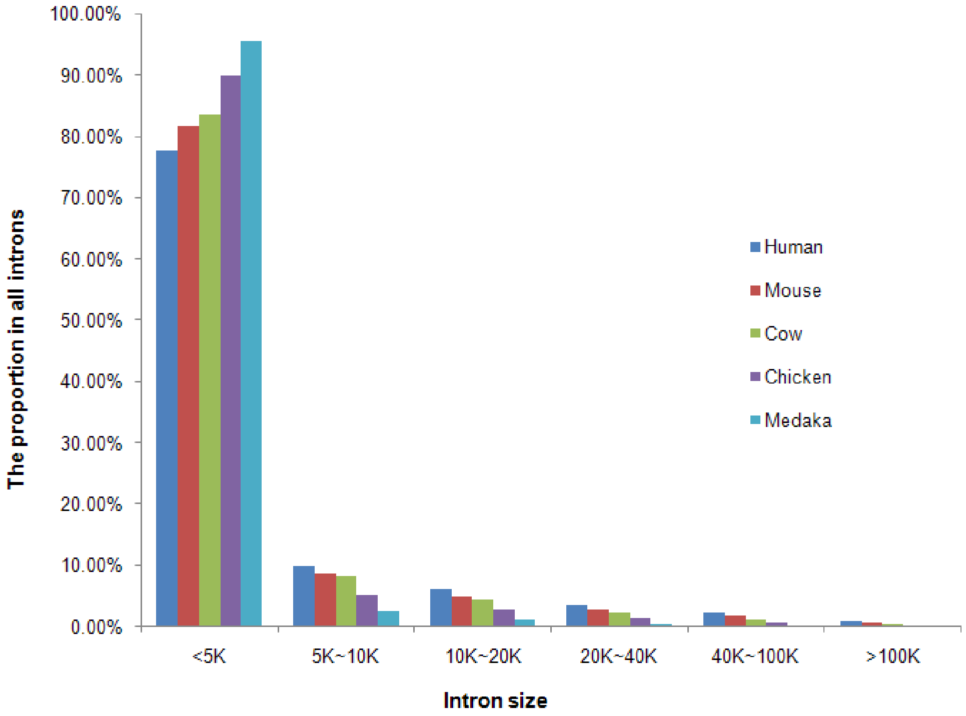

Five well-assembled genomes of different sizes from 0.7 Gb to 3.2 Gb are selected to estimate the distribution of large introns (longer than 5kb). The positions of introns in genomes are downloaded from Ensembl and the intron size is calculated as the difference value between start coordinate and end coordinate. Most introns are smaller than 5 kb. Although the proportion of long intron is low, they occupy over half of all gene loci (**Supplementary Table 1**).

Supplementary Figure 2. Comparisons of the performance of L_RNA_scaffolder with different parameters.
(a)

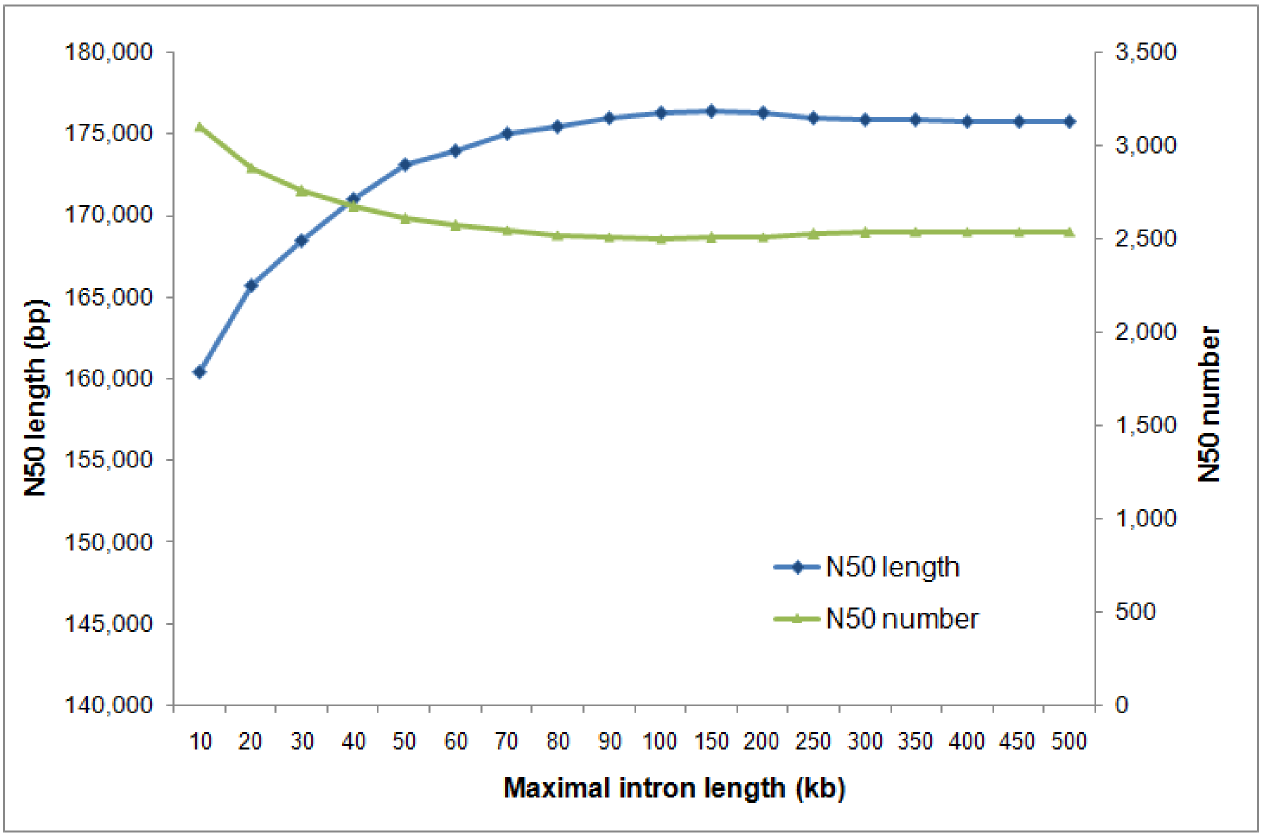

(b)

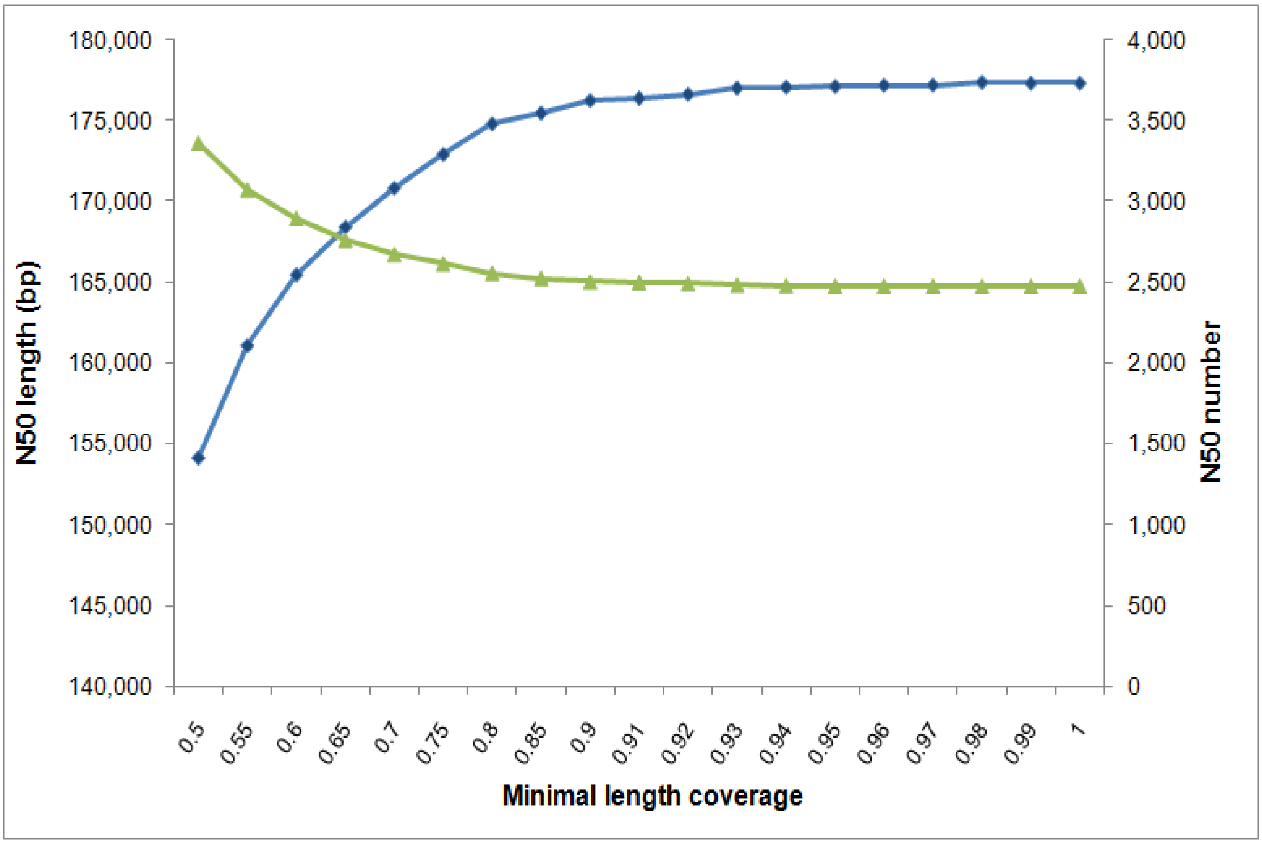

(c)

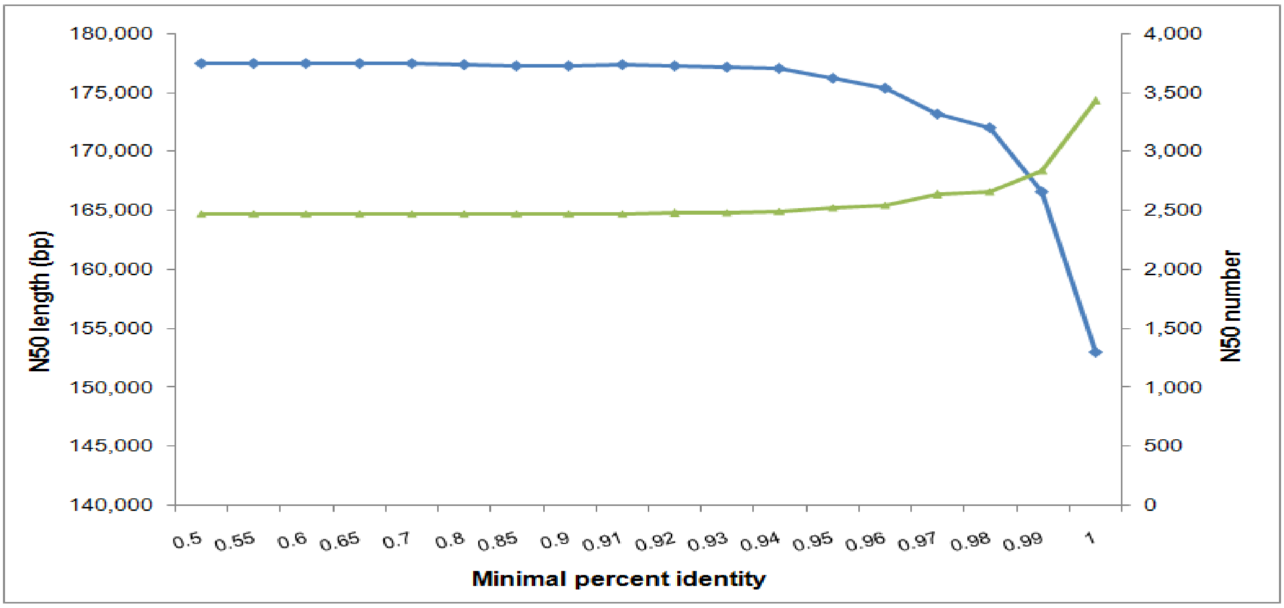

(a) The performance of L_RNA_scaffolder with minimal length coverage and minimal percent identity both set as 0.9. The N50 length is the length x such that 50% of the genome size is contained in sequences of length x or greater. The N50 number is the number of sequences with lengths greater than the N50 length.

(b) The performance of L_RNA_scaffolder with maximal intron length set as 100 kb and minimal percent identity set as 0.9.

(c) The performance of L_RNA_scaffolder with the maximal intron length set as 100 kb and the minimal length coverage set as 0.9.

Supplementary Figure 3. The contig number in zebrafish scaffolding paths.

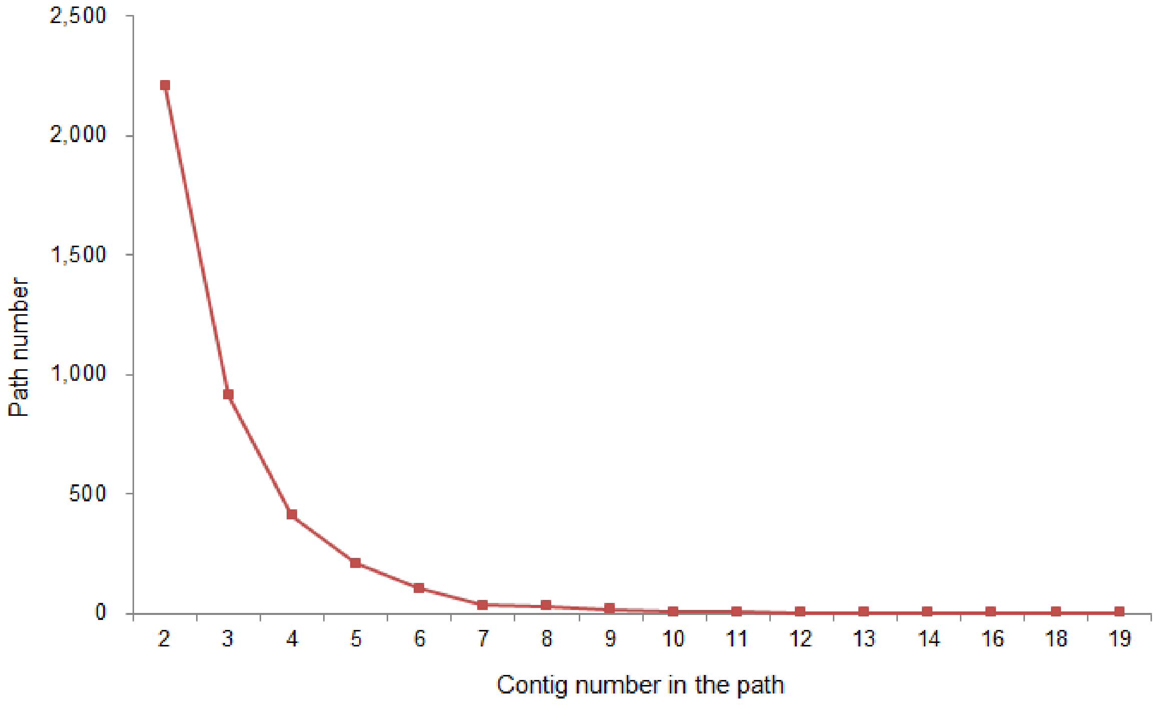

Every path starts from one predecessor and ends at one terminator. One path consists of at least two fragments.

Supplementary Figure 4. The length distribution of lost contigs in the correctable relocations.

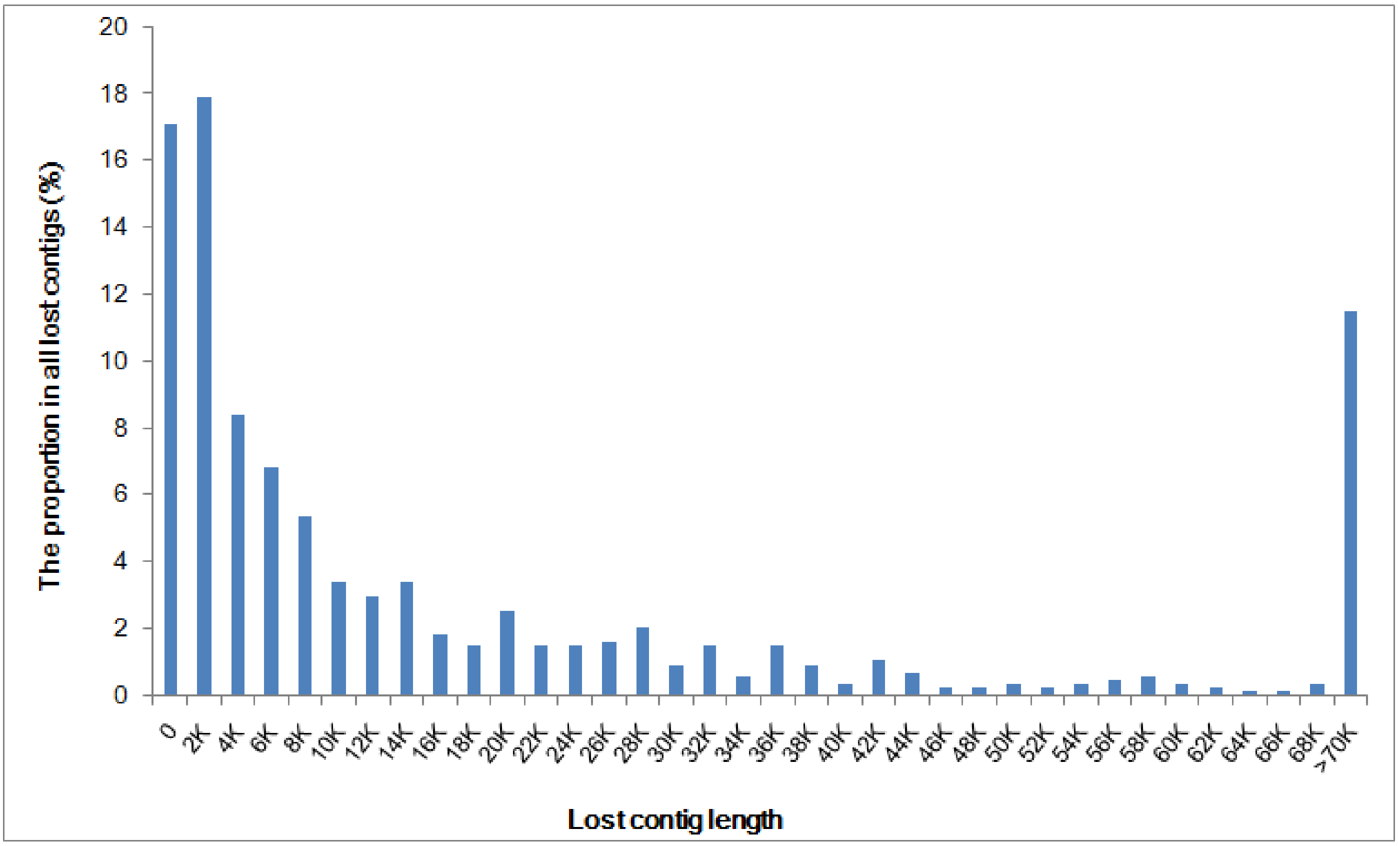

Supplementary Figure 5. The coverage of core eukaryotic genes in three versions of zebrafish genome

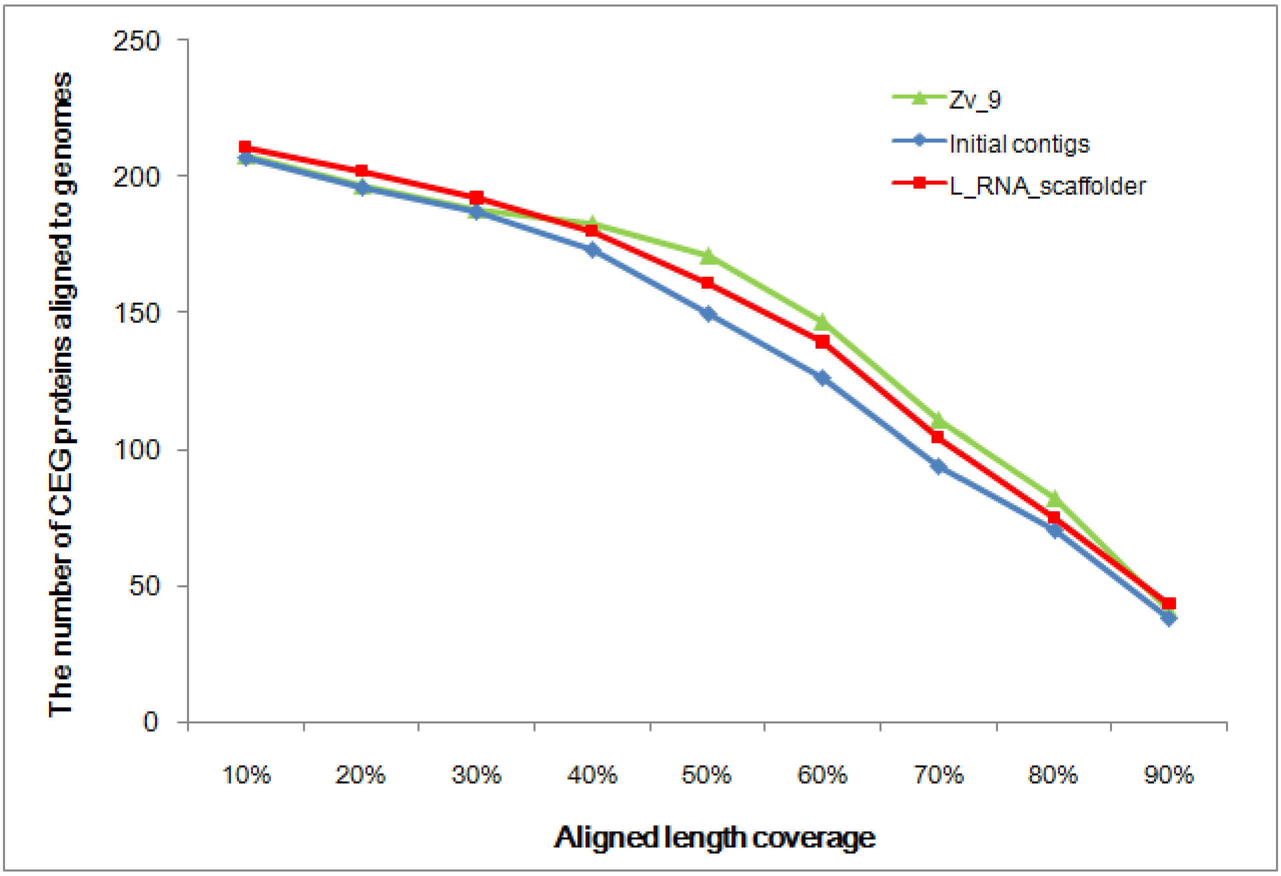

The 248 core eukaryotic genes aligned to three genomes of zebrafish using Blat with the default identity cutoff and different sequence coverage as shown on the x-axis.

Supplementary Figure 6. Alternative splicing may influence L_RNA_scaffolder accuracy

(a)

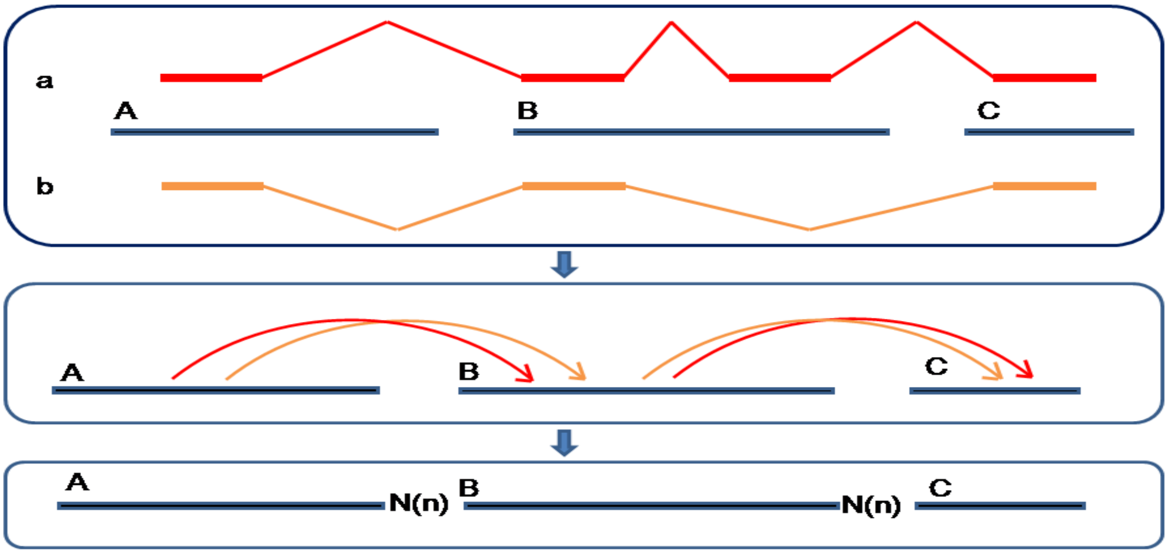

(b)

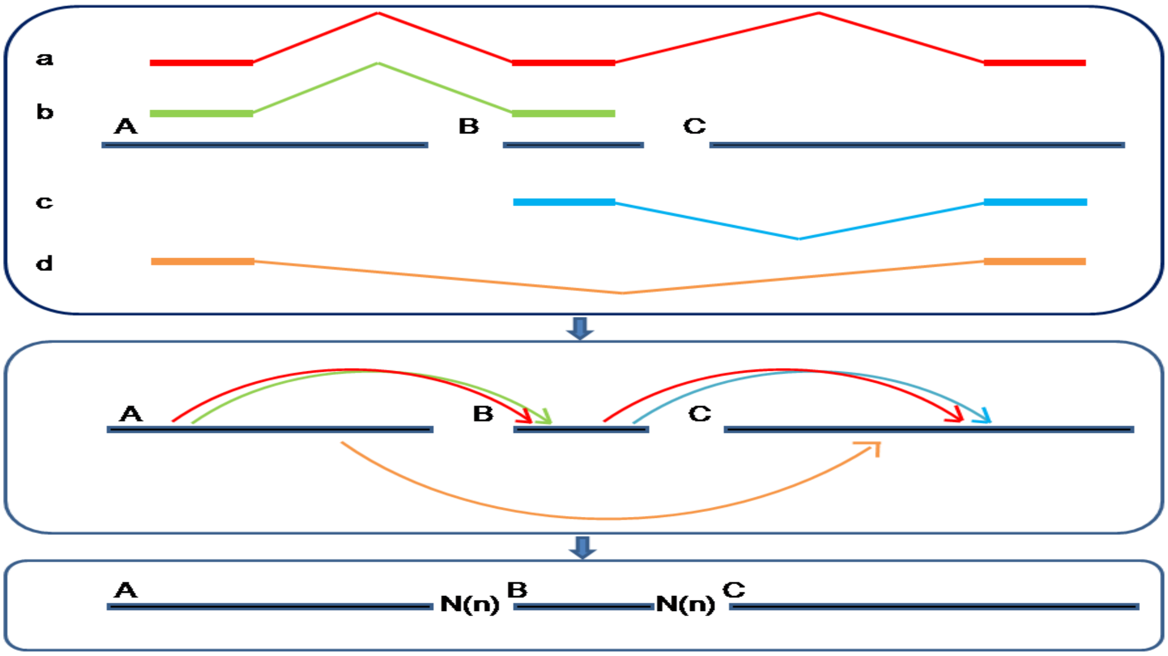

(c)

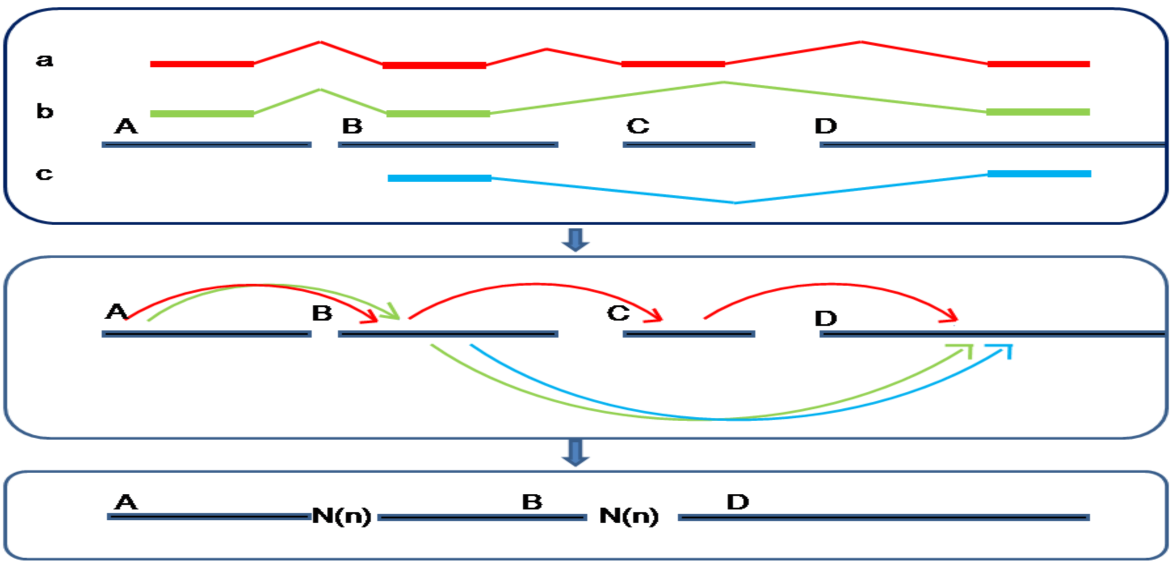

The A, B, C and D are the initial genome contigs. The reads a, b, c and d are alternative splicing transcripts aligned to these contigs.

(a) All spliced variants (a and b) are aligned to the same contigs (A, B and C). The built scaffolds could completely recover these transcripts.

(b) The dominantly expressed alternative splicing variant (a, b and c) includes all exons of this gene. They guide scaffolding and all variants could be also completely reconstructed.

(c) The dominantly expressed alternative splicing variant (b and c) includes partial exons of this gene. Some exonic contigs are not assembled during scaffolding.

Supplementary Figure 7. *Trans*-splicing and gene fusion may influence L_RNA_scaffolder accuracy

(a)

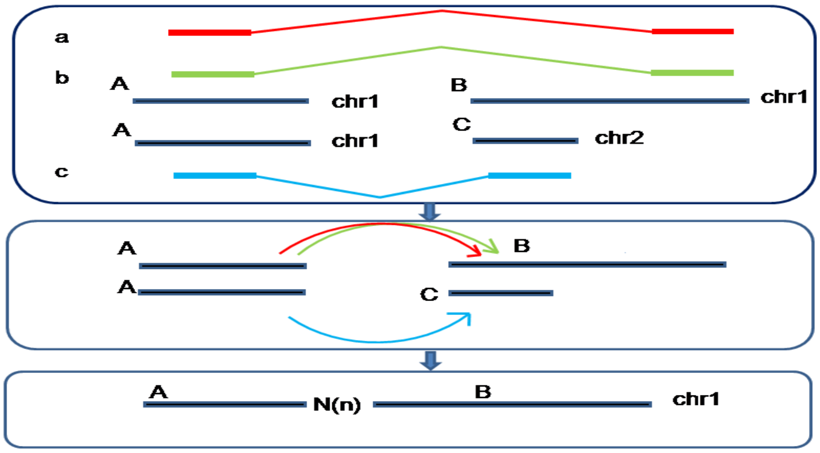

(b)

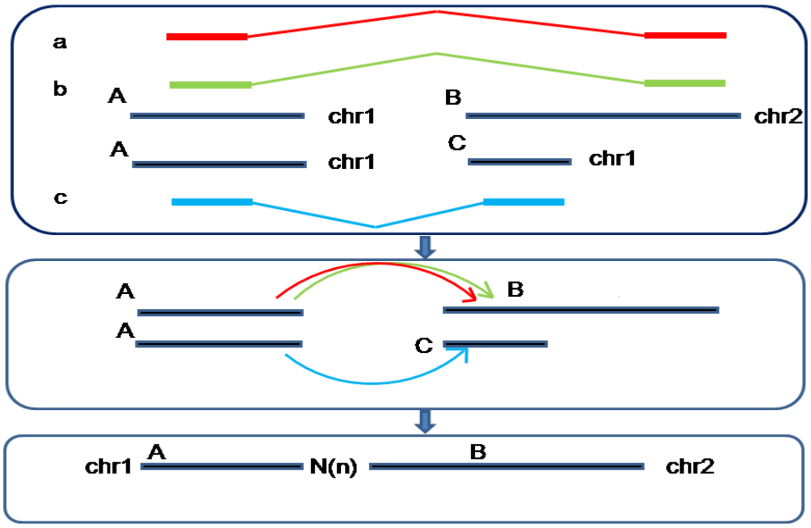

(a) The expression of one host gene in chromosome 1 (*a* and *b*) are higher than the chimeric RNA (*c*). Our method correctly rebuilds the genome under the guidance of host gene.

(b) The chimeric RNA has higher expression (*a* and *b*) than the host gene (*c*), leading to a translocation joining two contigs from chromosome 1 and chromosome 2.

Supplementary Tables

Supplementary Table 1. The proportion of long introns in transcribed genomic regions.

species	The proportion of gene loci in the whole genome [a]	Intron number	Intronic regions(bp)	The number of long introns (> 5kb)	Long intronic regions(bp)	The ratio of long intronic regions to the gene loci
human	1,566,452,199(bp)/3,287,209,763(bp)	353,969	1,502,476,963	93,511	1,314,618,765	83.92%
mouse	1,061,419,706(bp)/3,420,842,930(bp)	245,331	1,000,105,084	49,350	808,807,504	76.20%
cow	776,928,456(bp)/2,649,685,036(bp)	184,067	736,185,158	30,251	552,074,976	71.06%
chicken	413,418,146(bp)/1,050,947,331(bp)	156,204	384,899,057	15,454	252,841,527	61.16%
medaka	253,860,496(bp)/700,369,883(bp)	194,805	222,878,008	8,281	107,798,867	42.46%

[a.] The information analyzed here is all downloaded from Ensembl. The genes are firstly grouped into genomic loci based on their positions. If one genomic locus is mapped by only a single gene, then the length of this locus is the gene length. If one locus contains multiple overlapping genes in two strands, the length of this locus is the different value between the start coordinate of the first gene and the end coordinate of the last gene.

Supplementary Table 2. Twenty inversions in zebrafish genome scaffolding

The neighboring exons in 'guide' transcripts or homologs in the genome should have the same orientation. Therefore, 'guide' transcript and human homologs in zebrafish genome are used to determine the adjacent contigs orientation. For zebrafish 'guide' transcripts, “-“ indicates that the exon is aligned to the minus strand of the contig and “+” suggests that the exon is mapped to the plus strand of the contig. The mapping of human homologs to zebrafish contigs using chained tBLASTn, is downloaded from UCSC genome browser. “+-” shows that the exon is mapped to the minus strand of zebrafish contigs and “++” suggests that the exon is located in the plus strand. These 20 connections cases have the same order in L_RNA_scaffolder and Zv_9. But the orientation of one fragment is conflicting. Here, we show the contig order and orientation of these 20 cases in Zv_9. The transcript exon orientation and homolog orientation in Zv_9 contigs are listed below.

ID	contig in Zv_9 location [a]	contigs	guides	Exons [b]	Exons orientation in contigs [c]	Human homolog	Exons	Exons orientation in contigs
1	chr1:35053895-35093367:+	CABZ01031288.1	BC078294	233-380	-	NP_076981.2	80-129	+-
	chr1:35093468-35109701:+	CABZ01031289.1		377-687	+		129-231	++
2	chr1:54760837-54767651:+	CABZ01043818.1	EB930350	367-471	-	NP_000539.2	880-914	+-
	chr1:54767752-54810260:+	CABZ01043819.1		468-637	+		914-945	++
3	chr22:27050761-27155515:-	CR759791.15	CO810987	31-70	+	NP_001739.2	188-243	++
	chr22:27155516-27250314:-	CT954258.5		70-786	-		243-378	+-
4	chr17:47889492-47896557:-	CABZ01056332.1	DV586074	353-616	+			
	chr17:47695406-47889391:-	BX663528.10		613-748	-			
5	chr12:48266241-48267307:+	CABZ01091962.1	AF506733	3378-3716	+			
	chr12:48267408-48272500:+	CABZ01088984.1		3714-4549	-			
6	chr2:19557174-19691039:-	CU467620.11	AJ245962	0-684	+			
	chr2:19691040-19756295:+	CU928135.7		679-2045	+			
7	chr13:6075805-6169900:+	CU929430.10	BC092755	281-501	-			
	chr13:5922734-6075804:-	CR788289.5		497-1424	-			
8	chr1:59776395-59777648:+	CABZ01084505.1	BC046053	1196-1332	+			
	chr1:59777749-59778887:+	CABZ01083547.1		1355-1650	-			
9	chr12:1148757-1164194:+	CABZ01080099.1	EB933921	0-97	-			
	chr12:1164295-1169518:+	CABZ01080100.1		95-218	+			

10	chr14:649434-651655:+	CABZ01081123.1	EE319966	305-449	+			
	chr14:636877-649333:+	CABZ01113978.1		447-802	-			
11	chr18:26273659-26277108:+	CABZ01036953.1	CN502362	294-375	-			
	chr18:26261905-26273558:+	CABZ01036952.1		375-627	+			
12	chr21:43575806-43577805:+	CABZ01068725.1	AB231587	1217-1358	+			
	chr21:43577906-43582383:+	CABZ01067737.1		1358-1766	-			
13	chr1:12064599-12091344:+	FP340260.3	BC066445	0-512	+			
	chr1:12091345-12126855:+	CU550698.5		510-3566	-			
14	chr11:45206459-45210473:-	CABZ01080460.1	AY648803	0-199	+			
	chr11:45203995-45206358:+	CABZ01073275.1		192-470	+			
15	chr14:307840-309679:+	CABZ01088593.1	EE321901	174-248	-			
	chr14:309780-315541:+	CABZ01088594.1		274-436	+			
16	chr16:56669833-56685833:+	CABZ01053976.1	BC133154	163-1480	+			
	chr16:56668600-56669732:+	CABZ01096090.1		1467-1620	-			
17	chr17:53770525-53772992:-	CABZ01074990.1	CK142955	93-512	-			
	chr17:53768505-53770424:+	CABZ01107125.1		511-757	-			
18	chr2:52643770-52793870:-	BX248231.22	AL923663	0-172	+			
	chr2:52793971-52825316:-	CABZ01040021.1		172-491	-			
19	chr22:3249816-3257814:+	CABZ01084812.1	BC056722	432-1142	-			
	chr22:3245386-3249715:+	CABZ01084811.1		1139-1692	+			
20	chr9:3970864-3979864:-	CABZ01051291.1	EB932136	63-205	-			
	chr9:3944160-3970763:+	CU986282.4		203-646	-			

[a.] the format of “chr1:35053895-35093367:+” shows that contig “CABZ01031288.1” is located in the plus strand of chromosome 1 from the 35053895th nucleotide to the 35093367th nucleotide.

[b.] the format of “233-380” means that this exon starts from the 233rd nucleotide to the 380th nucleotide of the guide “C078294”.

[c.] The “-“ indicates that the exon of “233-380” is aligned to the minus strand of contig “CABZ01031288.1”. Since contig “CABZ01031288.1” is located at the plus strand of chromosome 1, this exon is aligned to the minus strand of chromosome 1. However, the exon of “377-687” is mapped to the plus strand of chromosome 1. The conflicting orientation of two adjacent exons suggests that one of two connected contigs should be reversed.

Supplementary Table 3. One hundred and two uncertain 'errant' relocations

Supplementary Table 4. Ninety eight 'errant' relocations where L_RNA_scaffolder connections had more supporting evidence

Supplementary Table 5. Twenty one 'errant' relocations where Zv_9 reference connections had more supporting evidence

Supplementary Table 6. Thirty three uncertain translocations

Supplementary Table 7. Forty three translocations where L_RNA_scaffolder connections had more supporting evidence

Supplementary Table 8. Sixteen translocations where Zv_9 reference connections had more supporting evidence

References

1. Salzberg SL, Phillippy AM, Zimin A, Puiu D, Magoc T, Koren S, Treangen TJ, Schatz MC, Delcher AL, Roberts M *et al*: **GAGE: A critical evaluation of genome assemblies and assembly algorithms**. *Genome Res* 2012, **22**(3):557-567.
2. Kurtz S, Phillippy A, Delcher AL, Smoot M, Shumway M, Antonescu C, Salzberg SL: **Versatile and open software for comparing large genomes**. *Genome Biol* 2004, **5**(2):R12.
3. **liftover** [http://genome.ucsc.edu/cgi-bin/hgLiftOver]
4. Huang S, Li R, Zhang Z, Li L, Gu X, Fan W, Lucas WJ, Wang X, Xie B, Ni P *et al*: **The genome of the cucumber, Cucumis sativus L**. *Nat Genet* 2009, **41**(12):1275-1281.
5. Grabherr MG, Haas BJ, Yassour M, Levin JZ, Thompson DA, Amit I, Adiconis X, Fan L, Raychowdhury R, Zeng Q *et al*: **Full-length transcriptome assembly from RNA-Seq data without a reference genome**. *Nat Biotechnol* 2011, **29**(7):644-652.
6. Au KF, Underwood JG, Lee L, Wong WH: **Improving PacBio long read accuracy by short read alignment**. *PLoS One* 2012, **7**(10):e46679.
